# Identification of Poly-*N*-Acetyllactosamine-Carrying Glycoproteins from HL-60 Human Promyelocytic Leukemia Cells Using a Site-Specific Glycome Analysis Method, Glyco-RIDGE

**DOI:** 10.1007/s13361-018-1938-6

**Published:** 2018-04-19

**Authors:** Akira Togayachi, Azusa Tomioka, Mika Fujita, Masako Sukegawa, Erika Noro, Daisuke Takakura, Michiyo Miyazaki, Toshihide Shikanai, Hisashi Narimatsu, Hiroyuki Kaji

**Affiliations:** 1 Glycoscience & Glycotechnology Research Group, Biotechnology Research Institute for Drug Discovery, National Institute of Advanced Industrial Science & Technology, Tsukuba, Ibaraki 305-8568 Japan; 2 Project for utilizing glycans in the development of innovative drug discovery technologies, Japan Bioindustry Association (JBA), Hatchobori, Chuo-ku, Tokyo, 104-0032 Japan

**Keywords:** Mass spectrometry, Hydrophilic interaction chromatography, Poly-*N*-acetyllactosamine, Polylactosamine, Glycoprotein, Site-specific glycome analysis, Glycopeptide, Glycoproteome, Glycan heterogeneity

## Abstract

To elucidate the relationship between the protein function and the diversity and heterogeneity of glycans conjugated to the protein, glycosylation sites, glycan variation, and glycan proportions at each site of the glycoprotein must be analyzed. Glycopeptide-based structural analysis technology using mass spectrometry has been developed; however, complicated analyses of complex spectra obtained by multistage fragmentation are necessary, and sensitivity and throughput of the analyses are low. Therefore, we developed a liquid chromatography/mass spectrometry (MS)-based glycopeptide analysis method to reveal the site-specific glycome (Glycan heterogeneity-based Relational IDentification of Glycopeptide signals on Elution profile, Glyco-RIDGE). This method used accurate masses and retention times of glycopeptides, without requiring MS2, and could be applied to complex mixtures. To increase the number of identified peptide, fractionation of sample glycopeptides for reduction of sample complexity is required. Therefore, in this study, glycopeptides were fractionated into four fractions by hydrophilic interaction chromatography, and each fraction was analyzed using the Glyco-RIDGE method. As a result, many glycopeptides having long glycans were enriched in the highest hydrophilic fraction. Based on the monosaccharide composition, these glycans were thought to be poly-*N*-acetyllactosamine (polylactosamine [pLN]), and 31 pLN-carrier proteins were identified in HL-60 cells. Gene ontology enrichment analysis revealed that pLN carriers included many molecules related to signal transduction, receptors, and cell adhesion. Thus, these findings provided important insights into the analysis of the glycoproteome using our novel Glyco-RIDGE method.

**Electronic supplementary material:**

The online version of this article (10.1007/s13361-018-1938-6) contains supplementary material, which is available to authorized users.

**Graphical Abstract:**
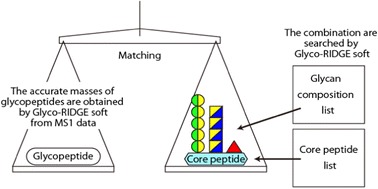
ᅟ

## Introduction

Protein glycosylation is a common type of post-translational modification involved in the folding, localization, stability, and interaction of proteins [1]. Therefore, elucidation of the relationship between protein action and glycosylation status requires analysis of glycosylation sites, glycan type (*N*- or *O*-linked), attached glycan structures and populations, and differences in glycosylation according to source cell type or cell status (physiological, stimulated, or disease) for individual glycoproteins of interest [2]. Considering the importance of the glycosylation status, it is necessary to analyze the target protein and compare the results for a number of coexisting proteins in the same sample. Accordingly, high-throughput analyses, e.g., omics analysis technology, are necessary. Comprehensive analysis of glycopeptides is thought to be a straightforward approach for such analyses but is technically difficult. Thus, glycopeptides were identified after removal of glycans, and the released glycans must be analyzed separately [3–6]. We therefore developed a method for large-scale identification of glycopeptides (peptide sequences and glycosylated sites) by glycopeptide capture, PNGase-mediated removal of glycans, and liquid chromatography (LC)/mass spectrometry (MS) analysis [7, 8]. This approach has facilitated the discovery of glycobiomarker candidates in hepatocellular carcinoma, lung adenocarcinoma, small cell lung carcinoma, and ovarian clear cell carcinoma [9–14] and has enabled the identification of glycosylated proteins from various organisms, including *Caenorhabditis elegans*, mice, and humans [15–19]. This knowledge was then used to construct a glycoprotein database, GlycoProtDB [16]. We also participated in studies of structural analysis of the released glycans in the HUPO international collaborative research activities of the Human Disease Glycomics/Proteome Initiative [20, 21]. Currently, the *N*-glycome released from the sample can be profiled rapidly by PNGase digestion, labeling for MS and fluorescence detection, purification, and LC/MS or LC/fluorescence analysis [22]. Profiling of *O*-glycans is still not easy [23]. In such separated analyses, information regarding the variety of glycans present at each site is lost. Accordingly, direct analysis of the glycopeptide is necessary to obtain the site-specific glycome.

Currently, large-scale analyses of glycopeptides are becoming possible; in particular, *O*-glycosylation sites can be identified by electron transfer dissociation (ETD) [24, 25] or electron-transfer/higher-energy collision dissociation (EThcD) [26] if the attached *O*-glycan is relatively small. EThcD is also effective for *N*-glycopeptide analysis [27]. However, large-scale analysis of *N*-glycopeptides is still difficult due to their intrinsic features, namely, the diversity and heterogeneity of attached glycans. This makes the acquisition and analysis of the MS2 spectrum of glycopeptides difficult. Moreover, these glycopeptides are composed of two types of oligomers, i.e., peptides and glycans, which are linked via peptide and glycoside bonds, respectively. Since these bonds have different energy levels, it is difficult to fragment the glycopeptides using a single dissociation method. Thus, identification of these glycopeptides requires tandem dissociation (MS2 or MS3) for overall fragmentation to identify both peptide sequences and glycan compositions [28–31]. For example, dissociation of an *N*-glycopeptide by collision-induced dissociation (CID) results in efficient cleavage of glycosidic bonds but leaves peptide bonds mostly intact. Therefore, identification of the peptide moiety requires further selection and dissociation of the peptide fragment (called the Y0 ion) or glycopeptide ion remaining the innermost GlcNAc of *N*-glycan (called the Y1 ion). Currently, no algorithm exists to selectively recognize these fragments among many fragment ions, which is the largest obstacle to the development of a method suitable to analyze glycopeptides automatically and comprehensively. Alternatively, ETD/electron capture dissociation cleaves peptide bonds without disrupting glycosidic bonds to enable peptide identification; unfortunately, this method is difficult to apply to glycopeptides because the mass of the attached glycan moiety is not presumable, and therefore, the mass gap corresponding to the modified residue cannot be assigned automatically. Notably, glycopeptide analysis using, for example, high-energy CID (HCD) and the combination of HCD and ETD has been developed, and search engines using the MS2 spectra of glycopeptides, such as Byonic and other analytical software, have emerged [32–45]. Furthermore, new measuring technologies, such as energy-resolved CID, ion mobility-resolved CID, and data-independent acquisition of MS2, have been developed, and the technical challenges of glycopeptide analysis have been overcome [46–49]. However, all current methods to identify both glycan composition and peptide sequence depend on dissociation of the target ion. Dissociation-based analysis methods exhibit decreased sensitivity. Sensitivity of the analysis is a critical issue for glycopeptide analysis because the ionization efficiency of glycopeptides is notably lower than that of nonglycosylated peptides. In addition, because glycans are heterogeneous, mass signals from a group of glycopeptides with a common peptide moiety are spread to multiple signals, resulting in lower overall signal intensities. Moreover, sialylation can increase glycan heterogeneity and further diminish signal intensity owing to the negative charge. Dramatic changes are needed to address this problem. Therefore, we designed an MS2-independent method for glycopeptide analysis. In principle, dissociation-dependent multistep measurements are less sensitive than MS2-independent methods, if the same mass spectrometer is used. The principle of the method was described previously [17]. Briefly, the method is comprised from two steps: (1) selection of MS1 signals of glycopeptides utilizing their glycan heterogeneity and chromatographic behaviors to obtain accurate masses and retention times on LC/MS analysis and (2) selection of combinations of core peptide and glycan compositions identified in advance from the same sample of glycopeptides by preexisting glycoproteomics (glycosylation site-mapping) and glycomics analyses. The accurate masses of glycopeptides must match the sum of the masses of the core peptides and glycans. By searching for combinations, glycopeptides can be assigned without MS2 analysis, which is critical to improve sensitivity.

Another cause of the lower sensitivity of MS analysis is ionization suppression. Notably, when minor components are ionized together with few major components, the signals of minor components cannot be detected, even if they are present in detectable amounts alone. Reducing the sample complexity by improving separation on LC during LC/MS or by prefractionating samples using a LC in diagonal mode, in contrast to the separation mode in LC/MS (reverse-phase LC), the detection rate of minor components is improved, and identification and detection of many components become possible.

Polylactosamine (pLN) glycans containing repeats of the *N*-acetyllactosamine (LacNAc) unit, (Galβ1-4GlcNAcβ1-3)n, are fundamental structures of glycans carried on *N*-glycans [50], *O*-glycans [51], and glycolipids [52]. pLN chains are further modified by the addition of different carbohydrate antigens, such as sialylation, fucosylation, and sulfation, thereby yielding functional carbohydrate antigens. As a result, pLN is known to function by presenting various glycan motifs, such as Lewis-type antigen and blood-type motifs, thereby mediating protein interactions, e.g., endogenous lectins, such as galectins [53], and some receptors, such as selectins [54]. pLN itself may be a ligand for cellular lectins, such as galectins [53]. The length of the pLN chain is an important factor in biological functions such as the immune response. For example, pLN inhibits natural killer (NK) cell-mediated cytotoxicity by inhibiting the cell binding process [55]. NK cells recognize and eliminate target cancer cells having sialyl Lewis x (sLe^x^) antigen on the short pLN chain of *N*-glycans [56]. Moreover, the glycosyltransferase *N*-acetylglucosaminyltransferase 5 (*MGAT5*) synthesizes the β1–6 branch of the *N*-glycan, yielding tetra-antennary *N*-glycans [57]; the synthesis of pLN is promoted on this β1–6 branch. T cells from *Mgat5*-deficient mice, which lack tetra-antennary *N*-glycans, have increased sensitivity to cell activation due to the lack of pLN-mediated surface interactions between the T-cell receptor complex and the galectin-3 [58]. Many biological functions of pLN have been identified in vitro; however, little is known of their functions in vivo*.* By analyzing *B2gnt2* gene-knockout mice lacking the major pLN synthase β3GnT2, which produces the pLN of *N*-glycan on glycoproteins [59, 60], we previously found that lack of long pLN chains on *N*-glycans results in enhanced immune responses [61]. Few studies have examined pLN-containing glycoproteins and investigated whether and how glycosylation is important for immunological function.

HL-60 cells, a human promyelocytic leukemic cell line, are capable of bidirectional differentiation into monocytoid and granulocytoid lineages [60, 62]. HL-60 cells are known to have pLN glycans on glycoproteins [63, 64] or glycolipids [65] and are used for investigation of the relationship between cell differentiation induction and glycans. Selectin ligands in HL-60 cells are present on pLN chains of *O*-glycans on some molecules, such as P-selectin glycoprotein ligand-1, and play important roles in lymphocyte homing [66]. However, few carrier glycoproteins carrying pLN chains on *N*-glycans have been identified.

To identify pLN carriers, a method for concentrating and identifying pLN carrier molecules using galectins [53] (e.g., galectin-1 or galectin-3), which bind to pLN chains, has been proposed. Because galectin-1 recognizes terminal LacNAc only, and galectin-3 recognizes both terminal LacNAc and the internal LacNAc structure, molecules containing a shorter lactosamine (LN) unit (LN < 3) are also enriched and identified using these galectins [67]. These methods using galectins are inefficient for identification of pLN carrier molecules containing long pLN chains.

Therefore, in this study, we attempted to construct an enrichment method for pLN carriers that were not influenced by the specificity and affinity of galectins to clarify the biofunctional role of pLN chains. We used hydrophilic interaction chromatography (HILIC) on an amide-80 column to separate glycopeptides based on their overall hydrophilicity. This separation is expected to improve the detection of minor signals, e.g., highly branched, highly fucosylated, or highly extended glycans (i.e., pLN).

## Experimental

**Figure 1:**
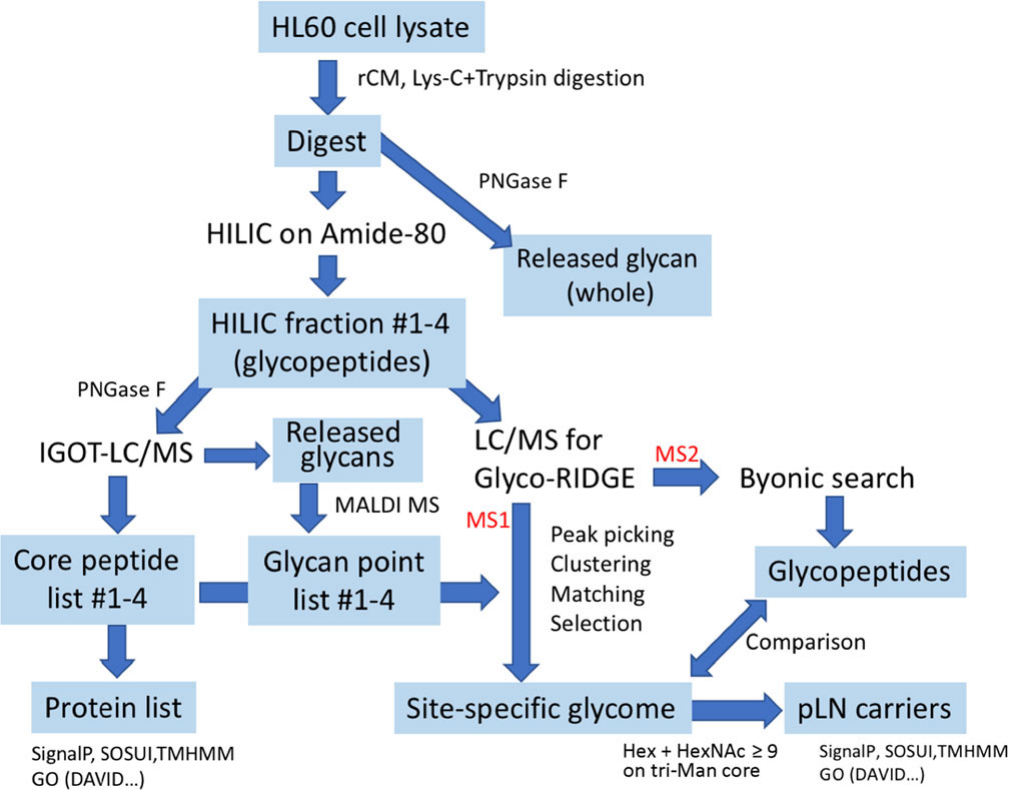
Strategy for site-specific glycan analysis by the Glyco-RIDGE method

The approach used in this study is summarized in Figure 1.

### Preparation and Fractionation of Glycopeptides

HL-60 human promyelocytic leukemia cells (#JCRB0085; Japanese Collection of Research Bioresources [JCRB] Cell Bank, Osaka, Japan) were cultivated as described previously [60]. Solubilization, S-reduction, alkylation, and digestion of the cellular protein were carried out as described previously [8]. Briefly, the cells (wet weight ~ 150 mg) were dissolved in 0.5 M Tris–HCl buffer (pH 8.5) containing 7 M guanidine-HCl and 50 mM ethylenediaminetetraacetic acid by vortexing followed by sonication. In this solution, the protein was reduced with dithiothreitol (10 mg) and alkylated with iodoacetamide (25 mg). After dialysis against 10 mM Tris–HCl (pH 8.6), the protein was digested with lysyl endopeptidase (Lys-C) and trypsin. An aliquot of the digest was acidified with trifluoroacetic acid (TFA) to pH 2 and heated at 80 °C for 2 h to remove sialic acid. Then, the digest was applied to HILIC on a TSKgel amide-80 column (2 mm id × 50 mm; TOSOH, Japan) to collect glycopeptides. The digest was diluted with three volumes of MeCN (final 75%) containing 0.1% TFA, loaded on the column, and washed thoroughly with 75% MeCN/0.1% TFA to remove nonglycopeptides. Glycopeptides were then eluted with a gradient of 75–45% MeCN containing 0.1% TFA to fractionate glycopeptides by their hydrophilicity (the length of glycans). From the start of the gradient program, 50 fractions were collected every 1 min (flow rate 0.2 mL/min) while monitoring the absorbance at 215 nm.

### Identification of Glycopeptides and Preparation of the Core Peptide List of each of four Glycopeptide Fractions

Each glycopeptide fraction was evaporated, dissolved with 50 mM Tris–HCl buffer prepared with stable isotope-labeled water (H_2_^18^O) and treated with PNGase F at 37 °C for 16 h (the isotope-coded glycosylation site-specific tagging (IGOT) method [8]). The digest was acidified with 0.2% formic acid and loaded on a trap column (Monocap C18 for trap, 0.2 mm id × 50 mm; GL-Science, Japan) at 0.015 mL/min. After washing with 0.1% formic acid, the column was connected to a nanoflow LC system which was constructed with two pumps (Shimadzu LC 20AD). The labeled peptides were separated on a tip column (C18 column, 0.15 mm id × 100 mm; 3 μm particles; Nikkyo Technos, Japan) with 5–25% (105 min) plus 25–35% (35 min) MeCN (+ 0.1% formic acid) gradients (200 nL/min). The effluent was ionized by electrospray and introduced directly into a mass spectrometer (LTQ Orbitrap Velos ETD; Thermo Fisher Scientific, USA) [17] with a source voltage of 2.0 kV and a capillary temperature of 250 °C. The mass spectrometer was operated by data-dependent acquisition in positive mode. The MS1 was obtained with an Orbitrap analyzer at a resolution of 30,000 at *m/z* 400 (*m/z* 300–1500, locked at 445.120030), and HCD MS2 data for four intense signals were obtained with an Orbitrap analyzer at a resolution of 7500, normalized collision energy (NCE) of 35, and exclusion of 60 s. The raw data file was processed with Mascot Distiller software (ver.2.6; Matrix Science, USA) and converted to an mgf file. The mgf file was then applied to a database search using the Mascot search engine (ver.2.5; Matrix Science) and the UniProtKB database (downloaded on July 1, 2017; 20,215 entries for humans). The search conditions were as follows: enzyme, trypsin + Lys-C; maximum missed cleavages, 2; fixed modification, carbamidomethy (C); variable modifications, ammonia loss (N-term C [carbamidomethyl]), delta:H(1)N(−1) 18O(1)(N) [this is registered in Unimod. Correctly, delta:H(−1)N(−1)18O(1)], Gln > PyroGlu (N-term Q), oxidation (M); MS1 tolerance, 7 ppm; and MS2 tolerance, 0.1 Da. The results file (.dat) was exported as a csv file using Mascot and processed with Microsoft Excel. We selected peptides with a rank of 1 and a pep_exp (expectation value) of less than 0.05 as identified peptides and then peptides with delta:H(1)N(−1)18O(1)(N) modifications, namely deglycosylated and labeled with ^18^O, on only the *N*-glycosylation consensus sequence (sequon; Asn-Xaa-[Ser/Thr/Cys], Xaa ≠ Pro) for glycopeptide identification. The nonredundant results having the lowest peptide expectation value (pep_exp) were used to make a core peptide list for each fraction; then, in the list, the intensity value and retention time for each peptide were obtained from the signal with the highest intensity in the redundant identifications (due to repeated analyses and different charges). Custom-made protein sequence databases were constructed based on the proteins identified by these analyses (#1–4) to use glycopeptide identification by Byonic. To search *O*-glycosylation for PNGase F-treated peptides, variable modifications of *O*-glycosylation [ST] were added to the Mascot search conditions, namely, HexNAc(1), HexNAc(1)Hex(1), HexNAc(2)Hex(2), or HexNAc(2)Hex(2)Fuc(1) for each search.

### MS Analysis of Released Glycans and Preparation of a Glycan Point List

Release and derivatization of *N*-glycans were performed according to a previously reported method, with slight modifications [17]. Briefly, the whole glycome of HL-60 cells was obtained from the tryptic digest of the cells. After PNGase F treatment, the digest was acidified with acetic acid (pH 3) and applied to an OASIS HLB cartridge (Waters, MA, USA). The pass fractions were recovered as the glycan sample. The glycome of each HILIC fraction was recovered from a pass fraction on the trap column of the IGOT-treated sample for LC/MS peptide identification. The glycans were treated with 1 M ammonium bicarbonate to substitute incorporated ^18^O with amine. These glycan samples were then cleaned using a PGC cartridge (Hypersep Hypercarb; Thermo) and then permethylated. Modified glycans were recovered with a Sep-Pak C18 cartridge (Waters) and analyzed with a matrix-assisted laser desorption ionization time-of-flight (MALDI-TOF) mass spectrometer (Ultraflex; Bruker Daltonics) using 2,5-dihydroxybenzoic acid.

Signal assignment was performed with Flexanalysis 3.4 software (Bruker Daltonics) and evaluated by manual inspection. The assigned glycan compositions were listed in the glycan point list, where the point of observed composition was set to 1 and that of the well-observed composition present in the glycan biosynthesis pathway, but not detected in each sample, was 0.1. Additionally, compositions that could not be synthesized by the biosynthetic pathway were listed as unusual compositions, e.g., Hex(5)HexNAc(1). The lists of identified glycans were used for Byonic search as described later.

### MS Analysis of Glycopeptides for Site-Specific Glycome Analysis

An aliquot of each fraction of desialylated glycopeptides (#1–4) was separated by nanoflow LC under the same separation conditions as deglycosylated (IGOT) peptides at the same day and just after IGOT-sample analysis and was analyzed by MS with an LTQ-orbitrap Velos ETD. The MS measurement was performed in a data-dependent manner and in positive-ion mode. MS1 was acquired using an Orbitrap analyzer at a resolution of 100,000 at *m/z* 400 (440–2000, locked at 445.120030). Intense 2 ions were selected and dissociated by HCD (NCE 35) for analysis of their MS2 spectra with Orbitrap (resolution 7500, exclusion 60 s). Using the MS1 data, monoisotopic mass, the highest intensity, and retention time at peak of all signals were chosen to generate a peak list with in-house software, named Glycan heterogeneity-based Relational IDentification of Glycopeptide signals on Elution profile (Glyco-RIDGE).

### Selection of Glycopeptide Signal from MS1 Data as a Cluster

To select glycopeptide signals from LC/MS data without MS2 information, we utilized two features of glycopeptides: the microheterogeneity of glycans and their chromatographic behaviors on reverse-phase LC. Specifically, natural glycopeptides must have heterogeneous glycans and a group of glycopeptides having a common peptide but heterogeneous glycans have similar retention times, as described previously. [17]. Thus, we selected glycopeptide signals as clusters according to the following three conditions: (1) the difference in retention time between glycopeptide signals was less than 1.0 min, (2) the difference in mass between glycopeptide signals was the mass of a monosaccharide (Hex, HexNAc, or dHex) with slight measurement error (7 ppm), and (3) a glycopeptide cluster had at least four members. Prior to the cluster search, MS1 data acquired as the *m/z* of multivalent ions was processed using the xtract module in Xcalibur (ver. 2.2; Thermo Scientific) to generate a peak list of monovalent ions (*z* = 1). The cluster search was performed with our in-house program, Glyco-RIDGE, under the conditions described above.

### Matching the Observed Accurate Mass of a Glycopeptide and the Calculated Mass of a Peptide Plus a Glycan in the List and Selection of the Most Likely Match

The combination of a peptide and glycan composition was searched by matching the observed accurate mass of a glycopeptide and the calculated mass of a core peptide identified in advance for the same sample of glycopeptide mass analysis. Here, the mass difference of a peptide and glycopeptide must be the sum of masses of comprising the monosaccharides, as calculated in Eq. 1:

1 $$ \mathrm{M}\left(\mathrm{glycopeptide}\right)=\mathrm{M}\left(\mathrm{peptide}\right)+\mathrm{M}\left(\mathrm{Hex}\right)\times \mathrm{x}+\mathrm{M}\left(\mathrm{Hex}\mathrm{NAc}\right)\times \mathrm{y}+\mathrm{M}\left(\mathrm{dHex}\right)\times \mathrm{z} $$

In this study, we searched for a combination meeting the following conditions: (1) glycans composed of Hex, HexNAc, and dHex; (2) *x*, *y*, and *z* are integer numbers and their ranges are *x*, *y*: 2–12 and *z*: 0–4; and (3) the mass difference tolerance was less than 5 ppm. If multiple matches were found, we selected the most likely match according to the following parameters: (1) total points of matched glycan compositions in the cluster, calculated using the glycan point list for each fraction; (2) intensity order of the core peptide list (stronger signals had smaller numbers); (3) retention time difference between the glycopeptide and core (deglycosylated) peptide (the core peptide was often eluted in the range of the retention time of its glycopeptide + 0–20 min); and (4) more strict mass difference (less than 2 ppm). If MS2-based search results of the member(s) or manual inspection of MS2 spectrum were available, we selected the likely combination according to the available information. For example, in cases in which a cluster had four members for peptide A in Fr #1, their assigned glycans were 620, 720, 820, and 920, respectively, and the total points in the cluster were four according to the point list of glycans. If, for the same cluster, different core peptide B molecules and different glycans were assigned, such as 331(1), 431(1), 531(0.1), and 631(0), then the total points were 2.1. In this case, the cluster on peptide A was selected. If the total points of the candidates were the same or close, and one assigned core peptide had core peptide no. 5, whereas another had core peptide no. 30, the former was selected.

### Assignment of Glycopeptide by MS2 Analysis and Byonic Search

The MS2 data set of glycopeptides was searched against a custom-made protein sequence database according to the results of core peptide identification for each fraction based on the human UniProtKB database and a database of the *N*-glycome actually detected in each fraction using the Byonic search engine ver. 2.10 (Protein Metrics) integrated as a node into Proteome Discoverer ver. 2.2 (Thermo Scientific). The search conditions were as follows: enzyme, trypsin; maximum number of missed cleavages, 2; static modification, carbamidomethylation (C); dynamic modifications, Gln > pyroGlu (N-term Q) and oxidation (M); precursor mass tolerance, 5 ppm; fragment mass tolerance, 0.1 Da; and maximum number of *N*-glycosylations per peptide, 2. The MS2 data for each clustered glycopeptide signal (M(z = 1)) were found using an in-house program (made and kindly provided by Dr. Issaku Yamada, Noguchi Institute, Japan). We selected probable hits from the Byonic search results based on the following criteria: Search Engine Rank = 1; Bionic score > 200; and DeltaM [ppm]: − 5–5.

### Prediction and Characterization of pLN-Carrying Glycoproteins

Glycoproteins having glycan compositions of more than nine saccharides (Hex + HexNAc≥9) were predicted as those carrying poly-*N*-acetyllactosaminoglycans. The presence of signal peptides and transmembrane (TM) segments was predicted using SignalP (ver.4.1, http://www.cbs.dtu.dk/services/SignalP/), SOSUI (Batch; http://harrier.nagahama-i-bio.ac.jp/sosui/), and TMHMM Server (v. 2.0; http://www.cbs.dtu.dk/services/TMHMM/), respectively. First, the signal peptide was predicted for each protein. If a signal peptide could not be detected, the TM was predicted using the whole protein sequence. If the peptide sequence was predicted, the TM was predicted using the protein sequence without the signal sequence. The larger number in the TM prediction by SOSUI and TMHMM was used for categorization of proteins as soluble or membrane proteins.

To investigate pLN-carrying glycoproteins at the functional level, we used gene annotation enrichment tools. One of enrichment tools, Database for Annotation, Visualization and Integrated Discovery (DAVID, https://david.ncifcrf.gov) [68], is a functional annotation bioinformatics tool that was used to cluster the genes for pLN-carrying glycoproteins, according to Gene Ontology (GO) analysis. Enrichment analysis was performed using the default settings of DAVID. DAVID calculates the statistical significance of GO term categories (i.e., cellular components, biological processes, and molecular functions) with enrichment scores and a *p* value. DAVID indicates functional enrichment for target genes. This analysis enabled us to directly evaluate predefined gene sets and pathways based on a given gene list of pLN carriers. In addition, to investigate the possible network (interactions) between pLN carriers, protein interaction networks were analyzed using STRING (https://string-db.org/) [69]. Analysis was set to default and included full information for the STRING databases. Analysis was performed with whole genome information as background. From the results of functional enrichment, molecules belonging to the network derived from annotation clustering of GO terms were highlighted using different colors.

The expression of glycogenes related to glycan synthesis in HL-60 cells was analyzed by Genevestigator [70]. As data for analysis, GSE36133 (expression data from the Cancer Cell Line Encyclopedia) was used. From these data, inputs related to glycogenes, such as glycosyltransferase genes, were selected.

## Results and Discussion

### Preparation and Fractionation of Glycopeptides

**Figure 2:**
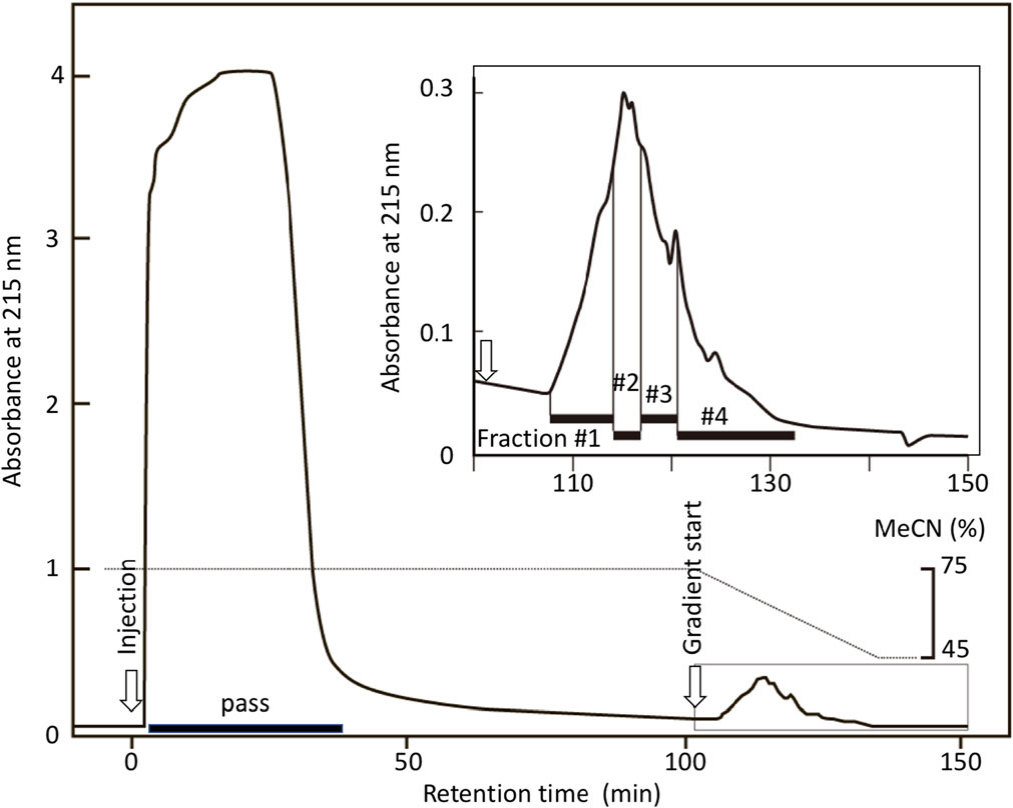
Chromatogram of HL-60 glycopeptides separated by HILIC on an amide-80 column. The tryptic digest of HL-60 proteins dissolved in 75% MeCN containing 0.1% TFA was applied to HILIC on an amide-80 column. After washing, adsorbed glycopeptides were eluted with a gradient of 75–45% MeCN containing 0.1% TFA. From the start of the gradient program, 50 fractions were collected every 1 min (flow rate 0.2 mL/min) by monitoring absorbance at 215 nm. They were combined into four fractions such that the absorbance areas were almost equal. Further analyses were carried out for each fraction

From 150 mg wet weight of HL-60 cells, protein was extracted, reduced, and alkylated. Approximately 9.9 mg protein was recovered, dialyzed, and digested with Lys-C and trypsin. The digest (2.5 mg equivalent, three runs) was diluted with three volumes of MeCN (final 75%) containing 0.1% TFA and loaded on an amide-80 column. After washing, bound glycopeptides were eluted with a 75–45% MeCN gradient and fractionated into 50 fractions (Figure 2). For fractions 5–31, the fractions were combined in four fractions such that the absorbance areas were almost equal; the fraction numbers were as follows: #1, fractions 5–11; #2, fractions 12–14; #3, fractions 15–18; and #4, fractions 19–31.

### Identification of Glycopeptides and Preparation of a Core Peptide List for Each of Four Glycopeptide Fractions

An aliquot (1/20) of each fraction (#1–#4) was treated according to the IGOT method (PNGase F treatment in H_2_^18^O) and analyzed by nanoflow LC/MS. From the four fractions, 352, 535, 942, and 797, glycopeptides were identified, respectively (Supplementary Tables S1–4; total of 1439 glycopeptides). In the pass fraction, few glycopeptides (deglycosylated peptides) were found by IGOT-LC/MS analysis (data not shown). Conversely, in the glycopeptide fractions (#1–4), small number of nonglycosylated peptides was identified. The percentages of glycopeptides in HILIC fractions were 94–97%, indicating that the preparation of glycopeptides by HILIC was successful (Supplementary Tables S1–4). For the identified sequence, the signal intensity and retention time at the highest peak intensity were retrieved from the .dat file to generate the core peptide list, wherein core peptide numbers were assigned in order of strong signal intensity for each fraction.

### MS Analysis of Released Glycans and Preparation of a Glycan Point List

Glycans from whole-cell lysates and each HILIC fraction were analyzed by MALDI-TOF/MS, and glycan compositions were assigned as described in Supplementary Table S5 and Supplementary Figures S1-1 to 1-11. The numbers of monosaccharides composed of glycans tended to be larger in fractions eluted later by HILIC fractionation. Fractionation of glycopeptides by HILIC is thought to depend on the overall hydrophilicity of the glycopeptide, not only the length (hydrophilicity) of the glycan. In fact, glycopeptides containing relatively short glycans were also contained in the hydrophilic fraction. The glycan composition of more than nine saccharides (Hex + HexNAc), expected to be pLN, was strongly observed in fraction #4. Therefore, the proportion of glycopeptides containing the long glycan (pLN) was lower in the total glycopeptide sample but enriched in fraction #4 by this fractionation method. Based on these results, glycan point lists, which were used for selection of the most likely match, were constructed as Supplementary Table S6.

### Selection of Glycopeptide Signals from MS1 Data as a Cluster

**Figure 3:**
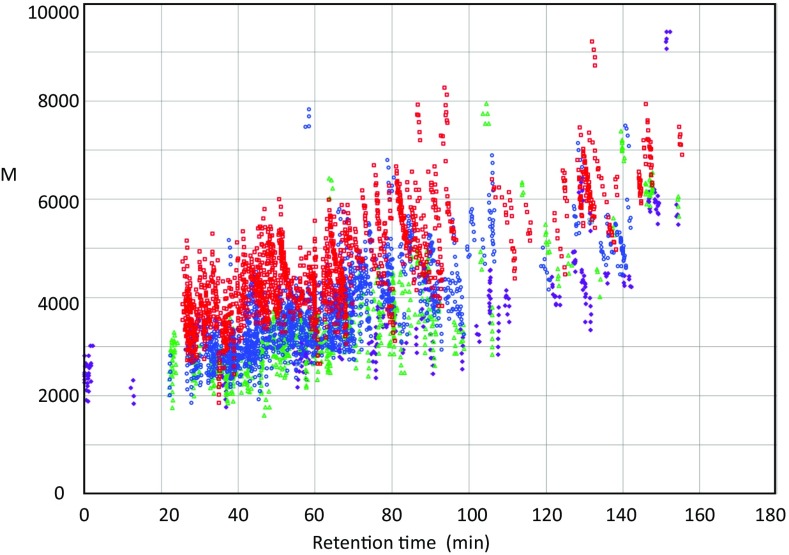
Retention time-M (mass) distributions of clustered signals. Clustered glycopeptide signals were plotted on a retention time-mass basis for each fraction. #1: purple, #2: green, #3: blue, #4: red. The distribution in the horizontal axis (mainly due to the degree of hydrophobicity of the peptide) did not differ largely from fraction to fraction; however, when the hydrophilicity of the fraction increased from fractions #1 to #4, the distribution in the mass direction showed a greater shift. This suggested that the more hydrophilic fractions contained longer attached glycans

We developed and reported a method to assign glycopeptides by matching the accurate mass of glycopeptides (observed) to a combination (sum) of the mass of the core peptide (calculated) and the mass of the glycan (calculated) [17]. A feature of this method is identification of glycopeptides without using the MS2 spectral information. According to this method, glycopeptide signals that were not selected for MS2 or were too weak to permit glycopeptide identification could be assigned as glycopeptide signals. As a result, the glycopeptide could be more efficiently and thoroughly identified. The strategy for this method was basically the same as that of a web-based tool, GlycoSpectrumScan, developed by Deshpande et al. [71]. The tool requires two types of inputs, oligosaccharide compositions present in the sample and in silico-derived peptide masses of a single protein of interest with a potential number of glycosylation sites, to fish glycopeptide signals from the MS spectrum. We extended the matching target to complex mixture of glycopeptides. If the matching was carried out by depending only on mass, many accidental matches would be obtained. Thus, to decrease the number of accidental matches, our method requires the collection of “accurate” masses of “glycopeptides” specifically from a huge number of signals in LC/MS data. A representative method to recognize that a signal is the signal of glycopeptide is to dissociate the signal by CID and then to check the resultant fragment ions are a series of diagnostic ions derived from a glycan, e.g., 366 (LacNAc), 204 (HexNAc), 186, 168, 144, or 138. However, with this data-dependent MS2 analysis, the number of confirmable precursor ions is limited. Therefore, we designed an additional method to fish and discriminate glycopeptide ions specifically by using two properties of glycopeptides (i.e., that the structure of the glycan attached to one attachment site was heterogeneous and that a group of glycopeptides having a common peptide portion and different glycan composition eluted at similar retention times on reverse-phase chromatography) instead of MS2. The types of monosaccharides constituting glycans are limited, and from the viewpoint of mass, the main components are limited to three species (Hex, HexNAc, and dHex), except for sialic acid. Difficulties related to the application of sialylated glycopeptides in our method will be discussed later. Thus, the mass difference between signals of glycopeptides with a common peptide corresponded to the mass of one or two monosaccharides. More specifically, the glycopeptide with a large glycan eluted earlier than the glycopeptide having a small glycan. Therefore, glycopeptide signals can be detected as signal clusters satisfying such elution conditions. In this study, we set the conditions to detect the glycopeptide signal cluster as follows: (1) the retention time difference between signals was 1 min or less, (2) the mass difference between signals corresponded to the above three glycan types (error 5 ppm), and (3) the minimum number of members in a single cluster was 4. To search clusters, we generated our in-house software named Glyco-RIDGE. MS1 data for each HILIC fraction were applied to the cluster search, and 57, 132, 218, and 196 clusters were detected in fractions #1–4, respectively (Table 1; Supplementary Tables S7–10). In total, 603 clusters composed of 4531 signals were collected (average number of members/cluster = 7.51). The average numbers of members/cluster for fractions #1–4 were 6.04, 6.68, 7.63, and 8.37, respectively (Table 1). The number increased as the hydrophilicity of the HILIC fraction increased. Clustered signals for each fraction were plotted by changing the color of the symbol (Figure 3). The range plotted against the retention time on the horizontal axis did not change significantly for each fraction. This indicated that the range of hydrophobicity of the peptide moiety did not vary much from fraction to fraction. However, when comparing the plot range of the mass in the longitudinal axis direction, the distribution was shifted to the high mass side as the degree of hydrophilicity of fraction increased. This indicated that the length of the glycan was longer as the fraction number increased from #1 to #4, consistent with the distribution of glycan compositions for each fraction (Supplementary Table S5).

**Table 1:** Summary of Glyco-RIDGE Matching and Byonic Search

HILIC Fr.	No. of detected cluster (A)	No. of GN-seq-comp combination (B)	No. of combination/cluster (= B/A)	No. of matched cluster by GR (C)	Percentage of match (= C/A%)	No. of GN-seq-comp combination matched (D)	Percentage of match (= D/B%)	No. identified by Byonic search (nr, S18–21)	No. of GR Hit identified by Byonic (nr)	No. of spectra searched by Byonic in GR member	No. of spectra showing the same core by GR and Byonic	Percentage of identity
#1	57	344	6.04	25	43.9	180	52.3	148	29	105	103	98.1
#2	132	882	6.68	54	40.9	384	43.5	229	57	91	91	100.0
#3	218	1664	7.63	67	30.7	579	34.8	340	109	134	132	98.5
#4	196	1641	8.37	54	27.6	589	35.9	71	16	23	23	100.0
Total	603	4531	7.51	200	33.2	1732	38.2	788	211	353	349	98.9

### Matching the Observed Accurate Mass of a Glycopeptide and the Calculated Mass of a Peptide Plus a Glycan in the List and Selection of the Most Likely Match

For each glycopeptide, the combination of its core peptide and glycan composition was searched in the list of core peptides and glycan compositions, which were categorized according to the number of monosaccharides (Hex 2–10, HexNAc 2–10 [12 for #4], and dHex 0–4 [5 for #4]). If multiple core peptides were matched, their total scores were compared, and the match with the larger score was typically selected. If the match was selected by the criteria (or single match), the total score of the cluster was indicated in the results (Supplementary Tables S11–14) with green shading. Most selections were carried out using this criterion. However, if in the matched glycan compositions, there was a considerable number of glycans with unusual compositions and no match, the matches were not selected. This was applicable for core peptides with a single match. If the total score was close, we selected or removed the match based on the criteria, such as the familiarity of the glycome (bias toward the biosynthetic pathway), delta mass (ppm; more strict mass accuracy), delta retention time (min), or core peptide number (order of signal intensity). If there were MS2-based identifications by Byonic or MS2 spectra applicable for manual inspection, we added the cluster to the final list. This selection was limited and aimed at collecting site-specific glycan information. Basically, all selections were performed based on the total score and other criteria, independent of Byonic results. Finally, we selected 25, 54, 67, and 54 clusters as the most likely matches for fractions #1–4, respectively (Table 1; Supplementary Tables S11–14); in total, 200 clusters composed of 1728 sequence-glycan combinations (average number of members/cluster = 8.64) were selected. The rate of selected clusters per detected cluster was 27.6–43.9% in each fraction (total 33.2%). This ratio was unexpectedly low. Based on this principle, the low selectivity was assumed to be caused by two factors: (1) that the core peptide was not present in the list and (2) that the ions in the detected glycopeptide clusters were not proton adducts. Indeed, short core peptides often could not be identified by LC/MS analysis of peptide moieties as IGOT peptides by a database search using Mascot. As described later, many core peptides of glycopeptides identified by MS2 analyses followed by Byonic search were not present in the core peptide list (Supplementary Tables S15–18; Supplementary Figures S2-1–2-5). In addition, clusters with high signal intensity were accompanied by adducts with ammonium, probably iron, or unknown ions (Supplementary Figure 3). There were some other problems, and although our analysis software is improving, cases with no matched peptides showed that elimination of false-positive matches using our current criteria was effective.

In this study, in the interval of the acquisition of MS1, a maximum of two MS2 spectra were measured in a data-dependent manner, and using these data, a database search was carried out in the Byonic search engine. Among 1728 matched glycopeptides, 211 had Byonic search results (a total of 353 spectra) with specified criteria of rank = 1 and Byonic score > 200. Among 353 spectra, 349 showed the same results by our MS1 matching method, i.e., Glyco-RIDGE (98.9% agreement; Supplementary Tables S11–14 and S15–18). This suggested that our selection criteria were effective. In addition, a large number of matches by the Glyco-RIDGE method without MS2 identification (1783–362 = 1421) was observed, indicating that the method was effective and showed high sensitivity and comprehensiveness. Conversely, there were many hits by Byonic search that were not detected by the Glyco-RIDGE method (577 of 788 peptide glycoforms; Supplementary Tables S15–18). These were found to have little glycan heterogeneity, with 1–3 glycan compositions/peptide. Among 19 glycopeptide groups having over four members (glycan compositions) found by Byonic, 16 groups could be found by the Glyco-RIDGE method. Many glycopeptides identified by only Byonic had high-mannose type glycans. There were only five compositions for high-mannose type glycans, i.e., 200–600 (200 shows a composition of Hex(2), HexNAc(0), and dHex(0) on trimannosyl core (Hex(3)HexNAc(2)). Four out of five glycopeptides having high-mannose must be detectable such that the glycoforms can be found as clusters by Glyco-RIDGE. If the lowest number of cluster members was changed to 3 from 4, more glycopeptides would be found as clusters. To recover these clusters, the software must be improved, and the search (matching) conditions should also be refined. The results of these analyses will be reported in future studies.

Finally, it should be pointed out that although the glycan compositions matched by the Glyco-RIDGE method are those of *N*-glycans, the core peptides identified by IGOT method might be partially *O*-glycosylated. Therefore, the presence of *O*-glycan was searched by Mascot for MS2 spectra of IGOT-peptides. As a result, six peptides were identified as *O*-glycopeptides (Supplementary Table S19). Among them, two were found in the matched tables, which suggests a possibility that these might be composed of both *N*- and *O*-glycans. Accordingly, their clusters are indicated in red in Supplementary Tables S11–14.

From the 1728 matched data, 1343 gene–site–glycan composition (GSC) matches were detected on 105 sites of 71 glycoproteins (Supplementary Table S20). In cases in which the GSC match was detected in multiple HILIC fractions, glycan length tended to be longer for the later (more hydrophilic) fractions. In this study, a total of 167 glycan compositions were matched (Supplementary Table S21). The most frequently matched glycans were high-mannose type, i.e., compositions 400, 500, 300, 600, and 200 (according to the composition on the trimannosyl core). Subsequently, the most frequently observed structure was complex-type glycans, particularly compositions at the early stage of the biosynthetic pathway, such as 221, 231, 331, 341, 441, 451, and 551. These data indicated that there was a high proportion of fucosylation in HL-60 cells. Furthermore, many glycans, including multiple fucoses, such as 222, 232, and 332, were observed, indicating the existence of fucose on the branch. FUT8, which is involved in the biosynthesis of the core fucose residue of *N*-glycans, was expressed at low levels in HL-60 cells (as shown in Supplementary Table S22). In addition, both FUT4 and FUT9, which synthesize internal Lewis x antigens and terminal Lewis x antigens on pLN chains, respectively [72], were also expressed in HL-60 cells. Since the expression of the *FUT4* gene was higher than that of the *FUT9* gene, the fucosylation (i.e., Lewis x epitope) was thought to be synthesized from the internal side (reducing end) of pLN chains.

As observed in the glycan mass spectra, glycans consisting of a relatively large number of monosaccharides, such as 561, 661, and 671, were found, particularly in fraction #4. Among the naturally occurring glycans, the composition in which the sum of Hex plus HexNAc is 9 or more, extension of the branch (pLN) is strongly suggested. The MS2 spectrum of one glycopeptide, peak 131 in cluster 4 of fraction #4, which was assigned as a glycopeptide of LAMP2 having glycan 661, showed glycan-derived fragment ions at *m/z* 138, 168, 204, and 366. In addition, ions at *m/z* 528 and 731 were observed, which were presumed to be Hex2HexNAc1 and Hex2HexNAc2, respectively, suggesting the presence of pLN extension (Supplementary Figure 4). Based on the expression of glycan-synthesizing enzyme genes (The Human Protein Atlas, https://www.proteinatlas.org/ENSG00000170340-B3GNT2/cell), the expression of *B3GNT2* (β3GnT2), which is responsible for the synthesis of pLN [59, 60], was high in HL-60 cells. In addition, many glycosyltransferases involved in the biosynthesis of pLN were expressed in HL-60 cells (Supplementary Table S22). HL-60 cells express the *B3GNT2* and *B3GNT8* genes, which encode the main enzymes involved in pLN synthesis on glycoproteins [73, 74]. In addition, the expression of *B4GALT1* (β4GalTI), which is involved in pLN synthesis, was also observed in HL-60 cells. When *B3GNT2* and *B3GNT8* are expressed at the same time, pLN synthesis activity is enhanced by cooperative action of both enzymes [73]. Gene expression of these *B3GNTs* is enhanced during cell differentiation into the myelocytic lineage [75]. In contrast, under the same conditions, no alterations in the expression of *B3GNT5*, which is involved in the synthesis of pLN on glycolipids, are observed [75]. This is consistent with the observation that pLN carriers are frequently found in this cell line [76].

### Identification and Characterization of pLN-Carrying Glycoproteins

Based on the glycan compositions of glycopeptides (Hex + HexNAc ≥ 9), putative pLN carrier proteins were selected; 31 proteins had such glycans (Table 2). Because 71 species of glycoproteins were matched by the Glyco-RIDGE method, about half (31 glycoproteins) were pLN carriers. This indicated that fractionation by HILIC was effective for enrichment and increased identification of pLN-carrying glycopeptides.

**Table 2:** Poly-*N*-Acetyllactosamine-Carrying Glycoproteins Assigned Based on Their Glycan Composition

	Entry name	Gene name	Protein description	Attached sites (sequon)	SignalP 4.1 (min15)		SOSUI + TMHMM	
					Signal peptide?	Position (new N-term)	No. of TM (max)	Category
1	PERM_HUMAN	*MPO*	Myeloperoxidase	323 (NIT)	Y	49	0	Soluble
2	SAP_HUMAN	*PSAP*	Prosaposin	80 (NAT), 101 (NMS)	Y	17	0	Soluble
3	RNAS2_HUMAN	*RNASE2*	Nonsecretory ribonuclease	86 (NMT), 111 (NLT), 119 (NIS)	Y	24	0	Soluble
4	CAP7_HUMAN	*AZU1*	Azurocidin	171 (NVT)	Y	20	0	Soluble
5	PRG2_HUMAN	*PRG2*	Bone marrow proteoglycan	86 (NLT)	Y	17	0	Soluble
6	CR1L_HUMAN	*CR1L*	Complement component receptor 1-like protein	437 (NYS)	Y	33	0	Soluble
7	CD97_HUMAN	*CD97*	CD97 antigen	38 (NAT), 453 (NNT)	Y	21	7	Membrane
8	S39A6_HUMAN	*SLC39A6*	Zinc transporter ZIP6	67 (NNS)	Y	21	6	Membrane
9	CD47_HUMAN	*CD47*	Leukocyte surface antigen CD47	111 (NYT)	Y	19	5	Membrane
10	PTPRC_HUMAN	*PTPRC*	Receptor-type tyrosine-protein phosphatase C	276 (NAS), 419 (NFT), 529 (NES)	Y	24	2	Membrane
11	CD33_HUMAN	*CD33*	Myeloid cell surface antigen CD33	209 (NLT)	Y	17	2	Membrane
12	CR1_HUMAN	*CR1*	Complement receptor type 1	578 (NYS)	Y	42	2	Membrane
13	LAMP1_HUMAN	*LAMP1*	Lysosome-associated membrane glycoprotein 1	84 (NTS), 249 (NTT), 261 (NKT)	Y	29	1	Membrane
14	ITB2_HUMAN	*ITGB2*	Integrin beta-2	501 (NNS)	Y	23	1	Membrane
15	SIGL5_HUMAN	*SIGLEC5*	Sialic acid-binding Ig-like lectin 5	210 (NLT)	Y	17	1	Membrane
16	CD44_HUMAN	*CD44*	CD44 antigen	57 (NST)	Y	21	1	Membrane
17	MPRD_HUMAN	*M6PR*	Cation-dependent mannose-6-phosphate receptor	83 (NHT)	Y	27	1	Membrane
18	BASI_HUMAN	*BSG*	Basigin	160 (NDS), 268 (NGS)	Y	19	1	Membrane
19	ITA4_HUMAN	*ITGA4*	Integrin alpha-4	480 (NRT), 626 (NCS)	Y	34	1	Membrane
20	NECT1_HUMAN	*NECTIN1*	Nectin-1	202 (NGT)	Y	31	1	Membrane
21	NPTN_HUMAN	*NPTN*	Neuroplastin	229 (NAT)	Y	29	1	Membrane
22	LMBD1_HUMAN	*LMBRD1*	Probable lysosomal cobalamin transporter	457 (NST)	N		10	Membrane
23	CD63_HUMAN	*CD63*	CD63 antigen	130 (NHT)	N		4	Membrane
24	CD53_HUMAN	*CD53*	Leukocyte surface antigen CD53	129 (NST)	N		4	Membrane
25	LAMP2_HUMAN	*LAMP2*	Lysosome-associated membrane glycoprotein 2	101 (NFT), 257 (NTT), 275 (NSS)	N		2	Membrane
26	TMED9_HUMAN	*TMED9*	Transmembrane emp24 domain-containing protein 9	125 (NST)	N		2	Membrane
27	AT1B3_HUMAN	*ATP1B3*	Sodium/potassium-transporting ATPase subunit beta-3	124 (NLT)	N		2	Membrane
28	AMPN_HUMAN	*ANPEP*	Aminopeptidase N	128 (NYT), 265 (NVT)	N		1	Membrane
29	LCAP_HUMAN	*LNPEP*	Leucyl-cystinyl aminopeptidase	834 (NCS)	N		1	Membrane
30	4F2_HUMAN	*SLC3A2*	4F2 cell-surface antigen heavy chain	365 (NIT)	N		1	Membrane
31	ERAP2_HUMAN	*ERAP2*	Endoplasmic reticulum aminopeptidase 2	714 (NIS)	N		0	Ambiguous

Glycoproteins containing pLN chains have been identified in previous studies. For example, basigin (CD147) has been reported to have pLN chains and sLe^X^ antigens at the nonreducing terminal of pLN [77]. This carbohydrate antigen functions as a ligand of E-selectin and is involved in neutrophil recruitment in renal ischemia/reperfusion [78]. Moreover, CD44 expressed in macrophages during follicular atresia contains pLN chains [79], and LAMP1 and LAMP2, which are expressed in lysosomes, are well-known pLN-rich proteins [80]. In GO term analysis of the pLN carrier molecules identified in this study, proteins were clustered in the “lysosome” category. Consistent with this, in a previous study, analysis of pLN on the LAMP protein of *Trypanosoma brucei* revealed that pLN chains on *N*-glycans functioned as sorting signals in endocytosis [81].

By estimating the transmembrane domain using prediction tools such as SOSUI and TMHMM, 25 of 31 species (81%) were presumed to be membrane proteins (Table 2). A similar prediction for the 514 proteins identified by the IGOT method (Supplementary Table S23) showed a similar proportion of membrane proteins (70%, 359/514); thus, pLN was not particularly characteristic of membrane proteins.

**Figure 4:**
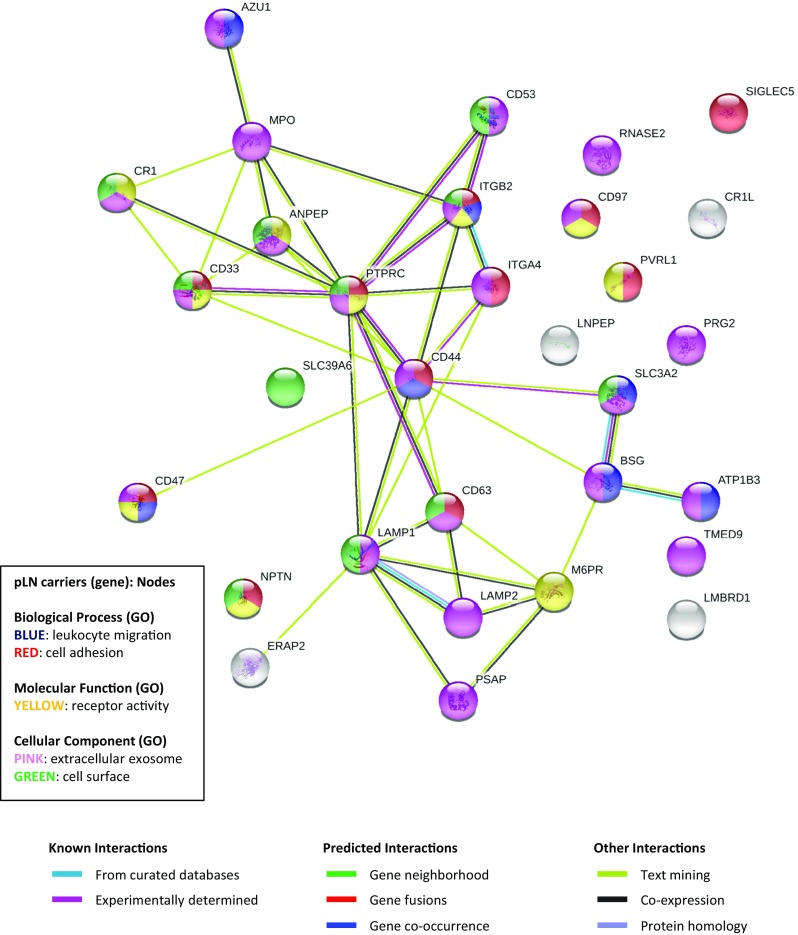
The illustration of the protein network analyzed using 31 pLN carriers. Protein network analysis of 31 pLN carriers was performed by STRING. Network nodes represented glycoproteins of pLN carriers. The color of the nodes indicates the query protein, which is related to the functional network of GO term categories. Enrichment analysis of 31 pLN carriers, performed by STRING, is shown in Supplementary Table S25

To confirm whether there were functional similarities among the 31 pLN carrier glycoproteins identified in this study, enrichment analysis by annotation clustering with GO terms was performed using DAVID (with DAVID default settings and species = *Homo sapiens*). As a result, as shown in Supplementary Table S24, nine annotation clusters were suggested. GO terms shown in clusters with higher enrichment scores (> 3) indicated the features of membrane glycoproteins, including signal (signal transduction), receptor, lysosome, and/or cell adhesion molecule. In addition, the results suggested that there was a cluster of molecules related to lysosomes containing molecules represented by known pLN carriers, such as LAMP1 and LAMP2. In the results of enrichment analysis based on only biological process (GO term BP-DIRECT) information, this category was enriched as a single cluster composed of the following GO terms: leukocyte migration (GO:0050900, count = 7 [23.3%], *p* = 4.3E−08, Benjamini = 1.02E−05), cell adhesion (GO:0007155, count = 7 [23.3%], *p* = 9.1E−05, Benjamini = 0.01), extracellular matrix organization (GO:0030198, count = 5 [16.7%], *p* = 3.0E−04, Benjamini = 0.03), and integrin-mediated signaling pathway (GO:0007229, count = 3 [10%], *p* = 1.2E−02, Benjamini = 0.38; enrichment score = 4.22) (data not shown). Additionally, the results of enrichment analysis were different due to differences in annotation tools (software) [82]. Therefore, additional GO term analysis and protein interaction network analysis were performed using STRING [69]. The protein network (relationship) of 31 pLN carrier molecules is shown in Figure 4. As shown in the results of annotation clustering (Supplementary Table S25), leukocyte migration (GO:0050900, count = 7 [22.6%], false discovery rate = 6.3E−5), cell adhesion (GO:0007155, count = 11 [35.5%], false discovery rate = 6.3E−5), and others were enriched in the biological processes category. In the molecular function category, receptor activity (GO:0004872, count = 10 [32.3%], false discovery rate = 3.0E−2) and others were enriched. Furthermore, in the cellular component category, extracellular exosomes (GO:0070062, count = 22 [71.0%], false discovery rate = 6.7E−11), cell surface (GO:0009986, count = 11 [35.5%], false discovery rate = 2.0E−7), and others were enriched. From our analysis of the features of pLN carrier glycoproteins, many molecules related to receptors on the cell surface, signal transduction, cell adhesion, and other categories were observed. Interaction between glycans (i.e., pLN carriers) and endogenous galectins has been reported to be involved in biological functions, e.g., cell adhesion [66]. This possibility should be investigated in further studies. Further analysis of secreted glycoproteins carrying pLN is also important to clarify the functions of pLN.

## Concluding Remarks

Direct analysis of glycopeptides is still technically difficult because glycopeptides have structures that are inherently challenging to analyze, i.e., glycan heterogeneity, differences in fragmentation energy between glycans and peptides, and low ionization efficiency. To overcome these barriers, we aimed to develop a method to identify glycopeptides without fragmentation, namely MS2. In this study, we applied our MS2-independent analysis method, named Glyco-RIDGE, to asialoglycopeptide fractions. Removal of sialic acid from the sample glycopeptides did not lead to inappropriate conclusions because the purpose of this study was to identify pLN carriers. Rather, desialylation was effective for this purpose. Namely, desialylation decreased the sample complexity and increased ionization efficiency. However, it is important to make this Glyco-RIDGE method applicable for analyzing the attachment state of sialic acid and its change at the proteomic scale. There are several disadvantages when attempting to use this method to analyze sialoglycopeptides. First, as described above, sialic acid is bound on various positions of the neutral glycan stem in different numbers. This dramatically increases glycopeptide species, resulting in a reduction in the abundance of individual glycopeptides, and decreases detection sensitivity. In addition, the negative charge of sialic acid suppresses the ionization efficiency. Another serious problem with this method is that sialylation alters (normally delays) the retention time of glycopeptides. The shift is larger than that by neutral saccharide addition and becomes larger depending on the number of sialic acid molecules, with a reverse direction. The shift caused by sialylation would be larger than the limit (~ 1 min) to search clusters of glycopeptides. According to our experience to date, asialo-, monosialo-, disialo-, and more sialylated glycopeptides were found as different clusters. This method utilizes the retention time difference between deglycosylated peptides (cores) and glycopeptide clusters to select likely matches. Currently, this is complicated by the larger shift resulting from sialylation. In our laboratory, we are now investigating the effects of sialylation on cluster distribution. We plan to establish a new criterion to connect sialoglycopeptide clusters and asialoglycopeptide clusters utilizing these behaviors.

As with desialylation, decreasing sample complexity is effective for increasing the number of glycopeptide signals. To achieve this, we separated glycopeptides by HILIC into four fractions by their hydrophilicity. From this analysis, 1439 sites on 514 proteins were identified by conventional IGOT-LC/MS analysis; among these sites, only 105 sites on 70 proteins were identified. In our glycopeptide analysis, only approximately 14% of proteins and 7.3% of sites yielded information, despite the use of approximately 20 times the amount of sample in site-mapping analysis. This was consistent with the difficulty of identifying glycopeptides. Of the 600 clusters composed of 4500 glycopeptide glycoforms detected, core-composition matches were made in 200 clusters with 1728 glycoforms matches, and the assignment rates were 32.5 and 39.4%, respectively. The cause of this low match rate must be addressed by improving the methods used to list core peptides, coping with adducts other than protons, and optimizing the minimum number of cluster members. Compared with the results of MS2-based analyses followed by Byonic searching, assignments of only 362 MS2 spectra were obtained among 1783 glycopeptides. The remaining 1517 glycopeptides (about 88%) were thought to be minor components that could not be selected for MS2 or their MS2 spectra were difficult to identify. Therefore, our MS1-based method is thought to be more sensitive. By using this method to complement other MS2-dependent analysis methods, the efficiency and coverage rate of glycopeptide analysis will be improved.

## Electronic Supplementary Material

ESM 1. Supplementary Figures S1-1 – 1-11. Mass spectra of permethylated glycans. Supplementary Figures S2-1 – 2-5. Representative MS2 spectra of glycopeptides identified and annotated by Byonic in each HILIC fraction. Supplementary Figure S3. Representative mass spectrum of glycopeptide. Proton adduct of a glycopeptide is accompanied with several adduct ions with ammonium, probably iron, and unknown ion. Supplementary Figure 4. MS2 spectrum of glycopeptide. In addition to well-known diagnostic ions of glycan (138, 168, 204, and 366), fragment ions of H2HN1 (528) and H2HN2 (731) were observed, suggesting the presence of pLN. (PDF 2532 kb)

ESM 2 (XLSX 1843 kb)
