# Supplementary material for: Identification of Poly-N-Acetyllactosamine-Carrying Glycoproteins from HL-60 Human Promyelocytic Leukemia Cells Using a Site-Specific Glycome Analysis Method, Glyco-RIDGE
Source: J Am Soc Mass Spectrom. 2018 Apr 19;29(6):1138–52. doi: 10.1007/s13361-018-1938-6 (PMC6004004; doi:10.1007/s13361-018-1938-6)
Supplement: Supplementary file 1 — Supplementary Figures S1-1 – 1-11. Mass spectra of permethylated glycans. Supplementary Figures S2-1 – 2-5. Representative MS2 spectra of glycopeptides identified and annotated by Byonic in each HILIC fraction. Supplementary Figure S3. Representative mass spectrum of glycopeptide. Proton adduct of a glycopeptide is accompanied with several adduct ions with ammonium, probably iron, and unknown ion. Supplementary Figure 4. MS2 spectrum of glycopeptide. In addition to well-known diagnostic ions of glycan (138, 168, 204, and 366), fragment ions of H2HN1 (528) and H2HN2 (731) were observed, suggesting the presence of pLN. (PDF 2532 kb) [file 13361_2018_1938_MOESM1_ESM.pdf]

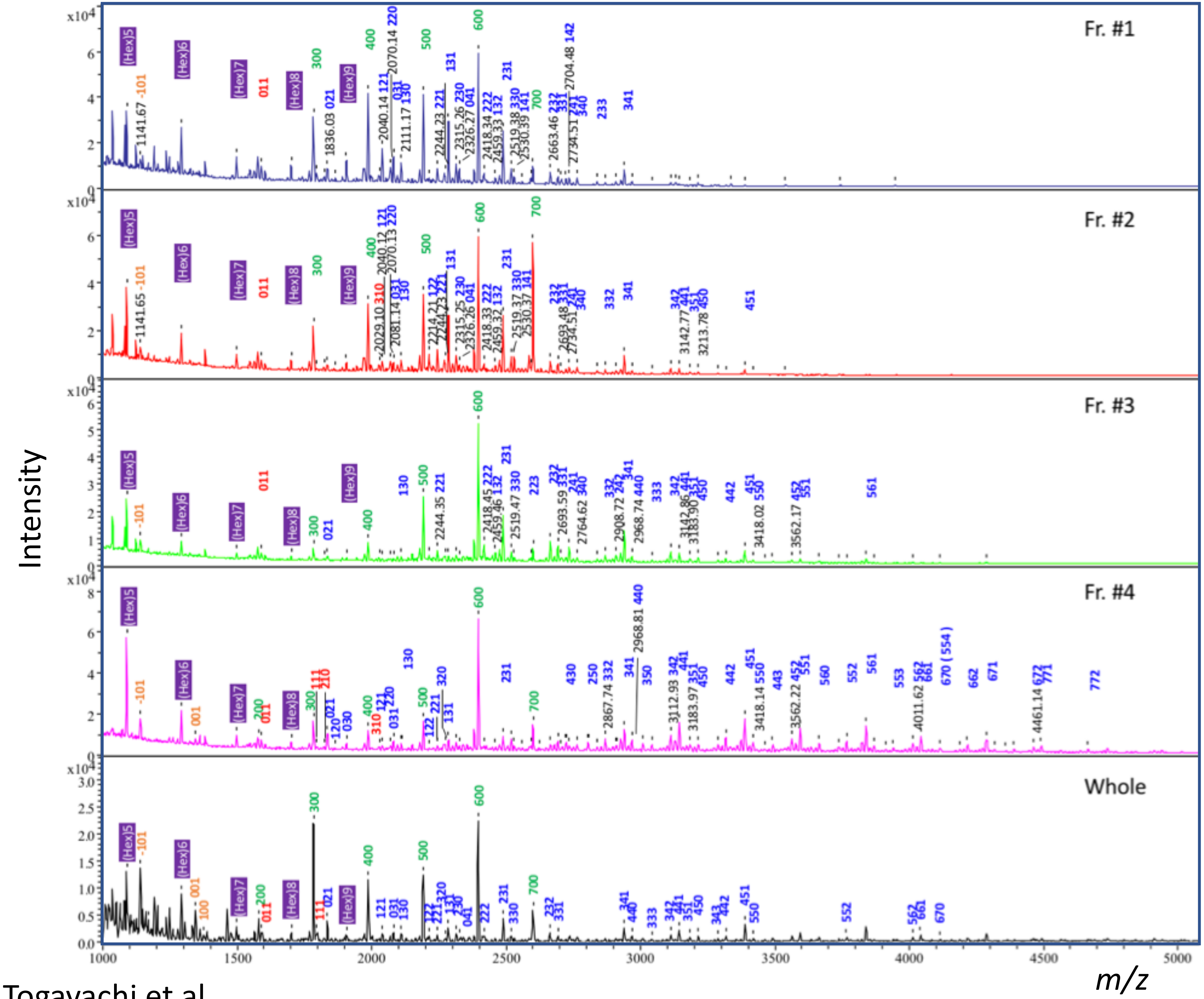

Fig. S1-1. Togayachi et al.

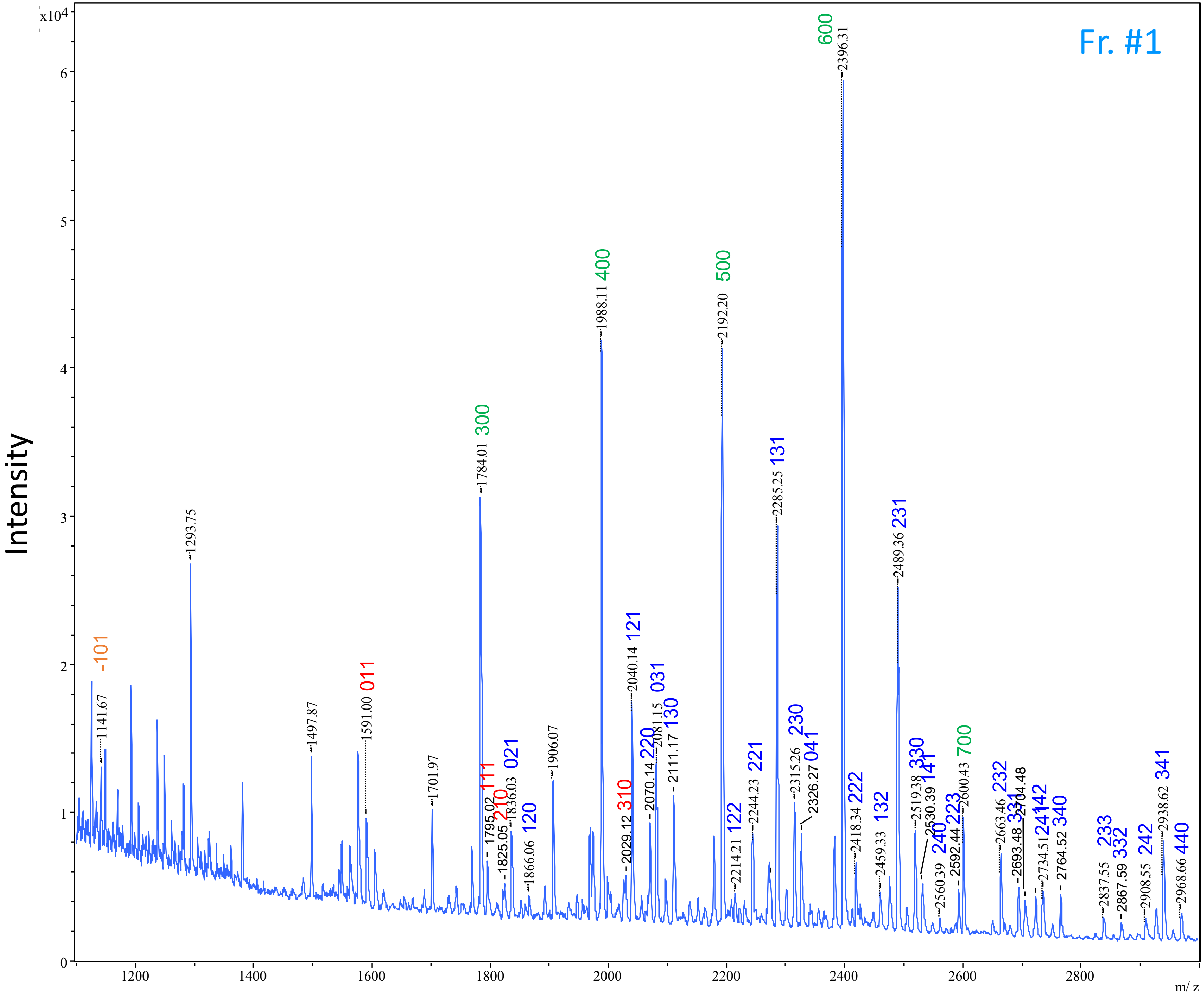

Fig. S1-2. Togayachi et al.

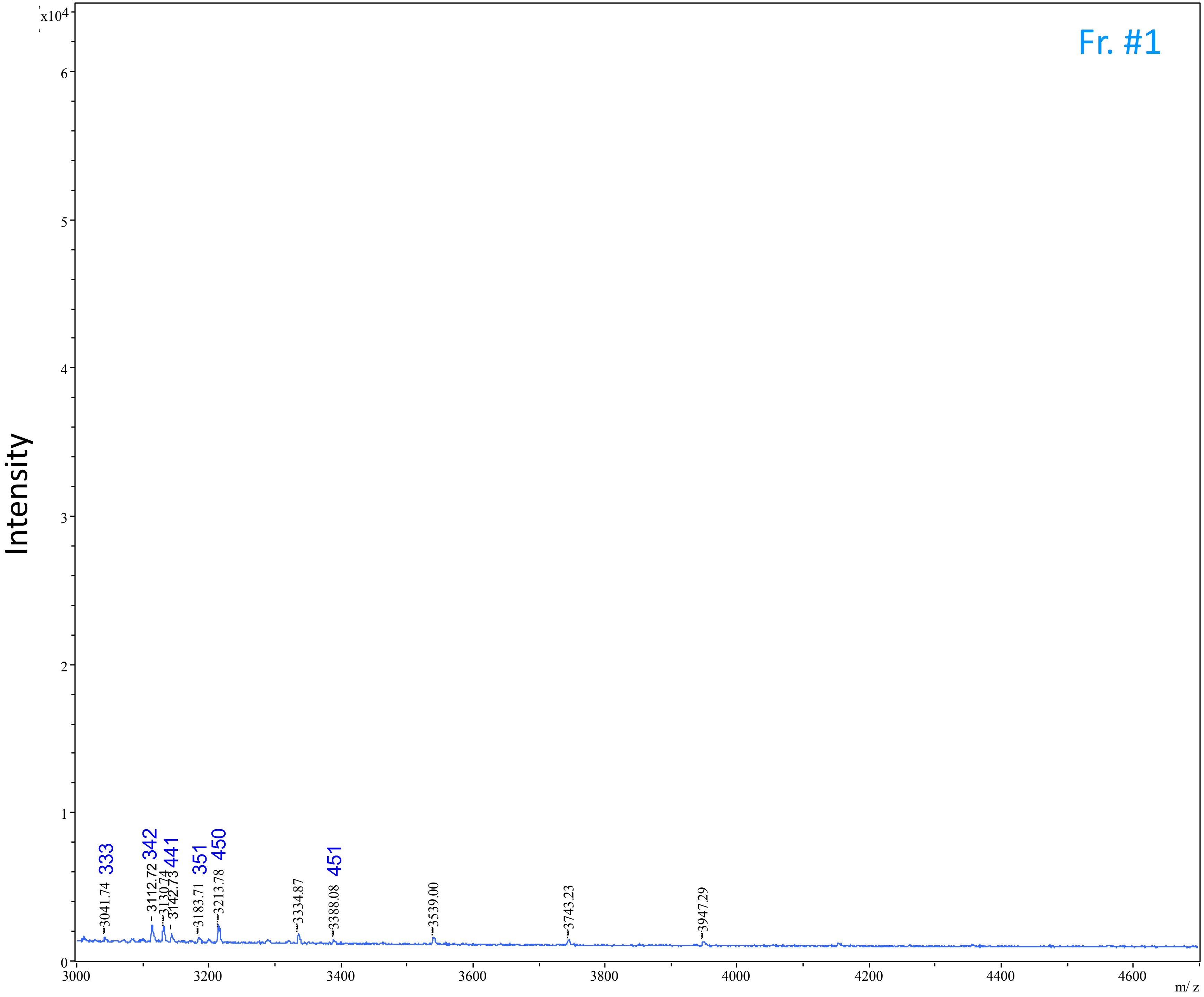

Fig. S1-3. Togayachi et al.

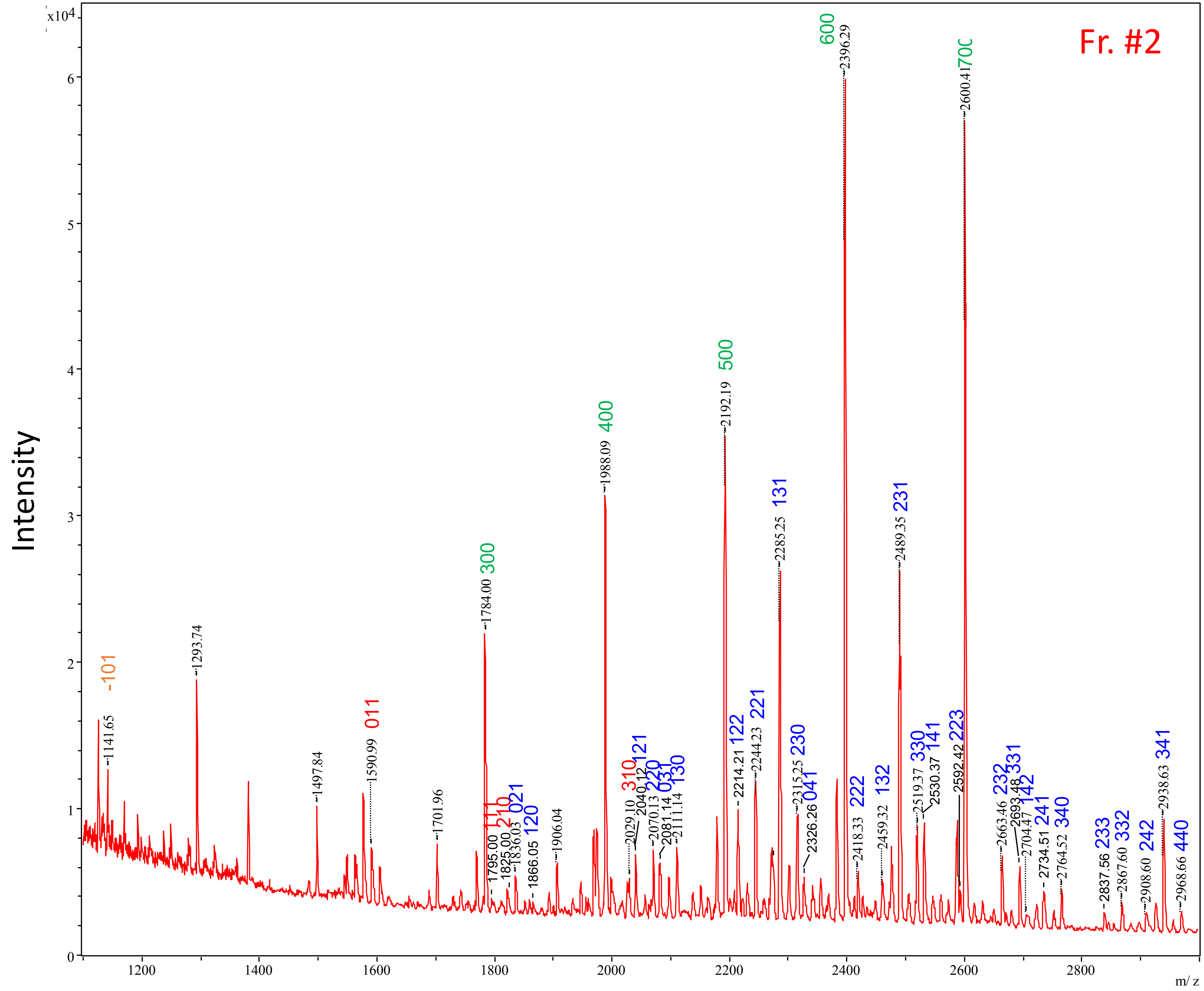

Fig. S1-4. Togayachi et al.

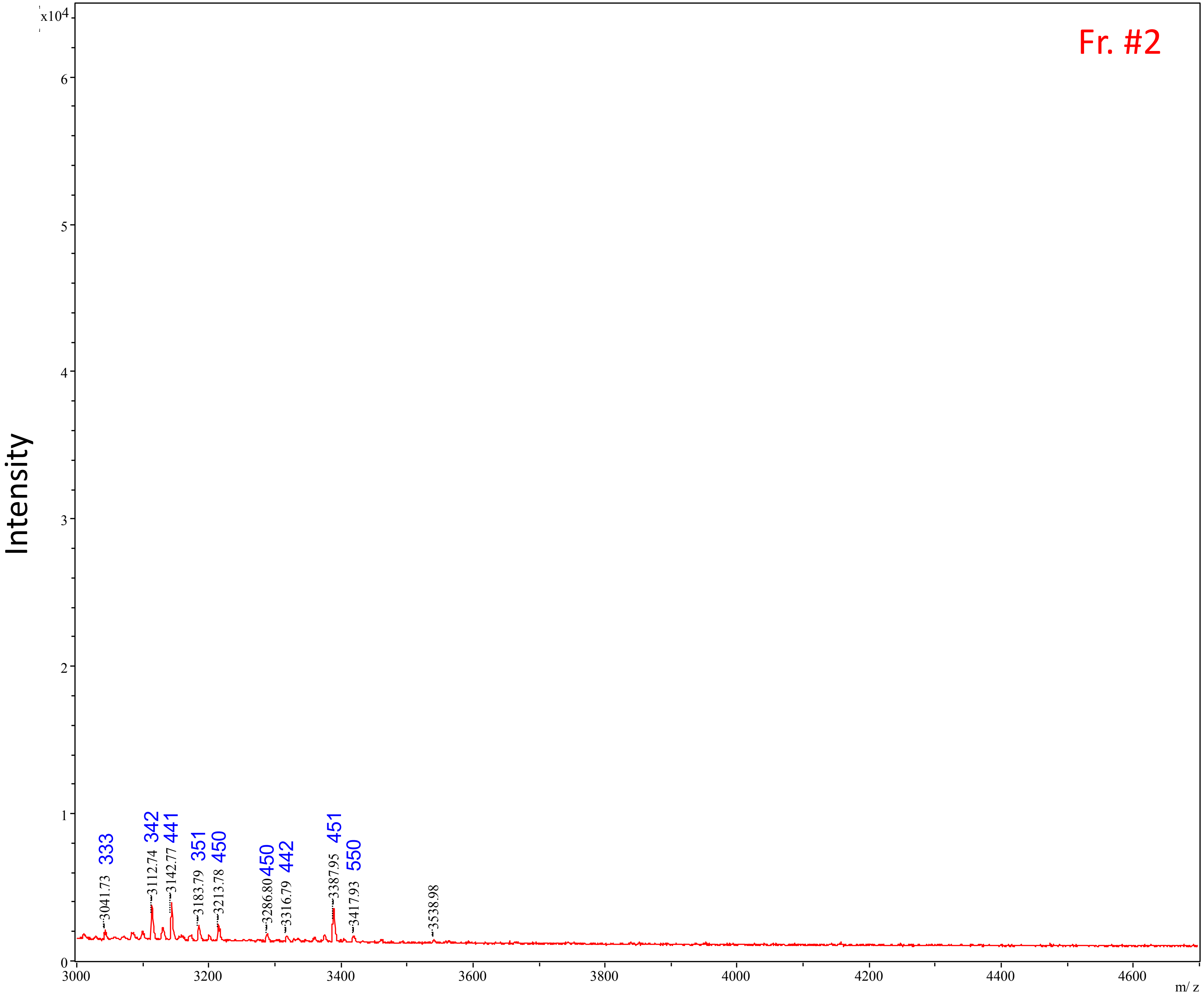

Fig. S1-5. Togayachi et al.

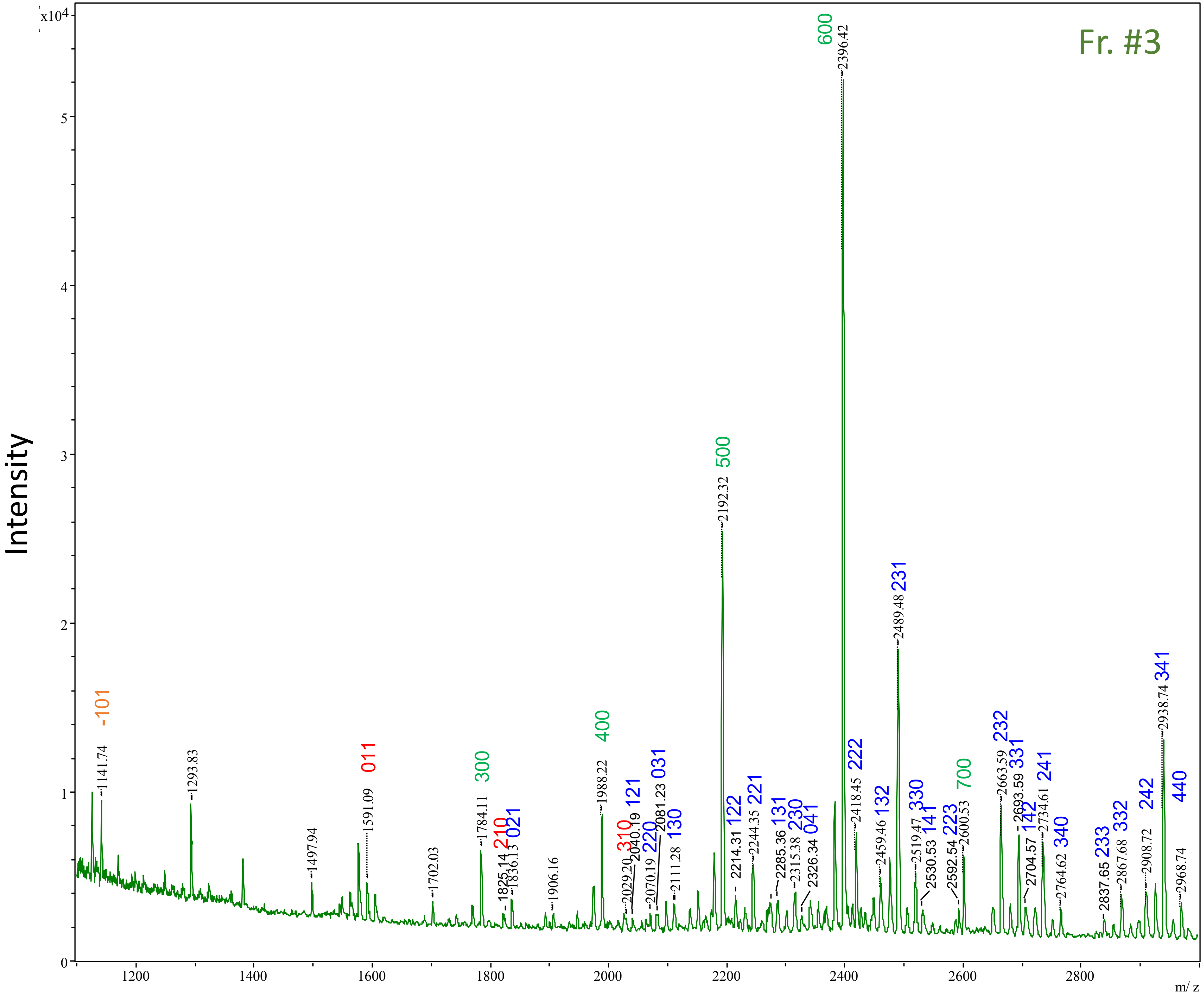

Fig. S1-6. Togayachi et al.

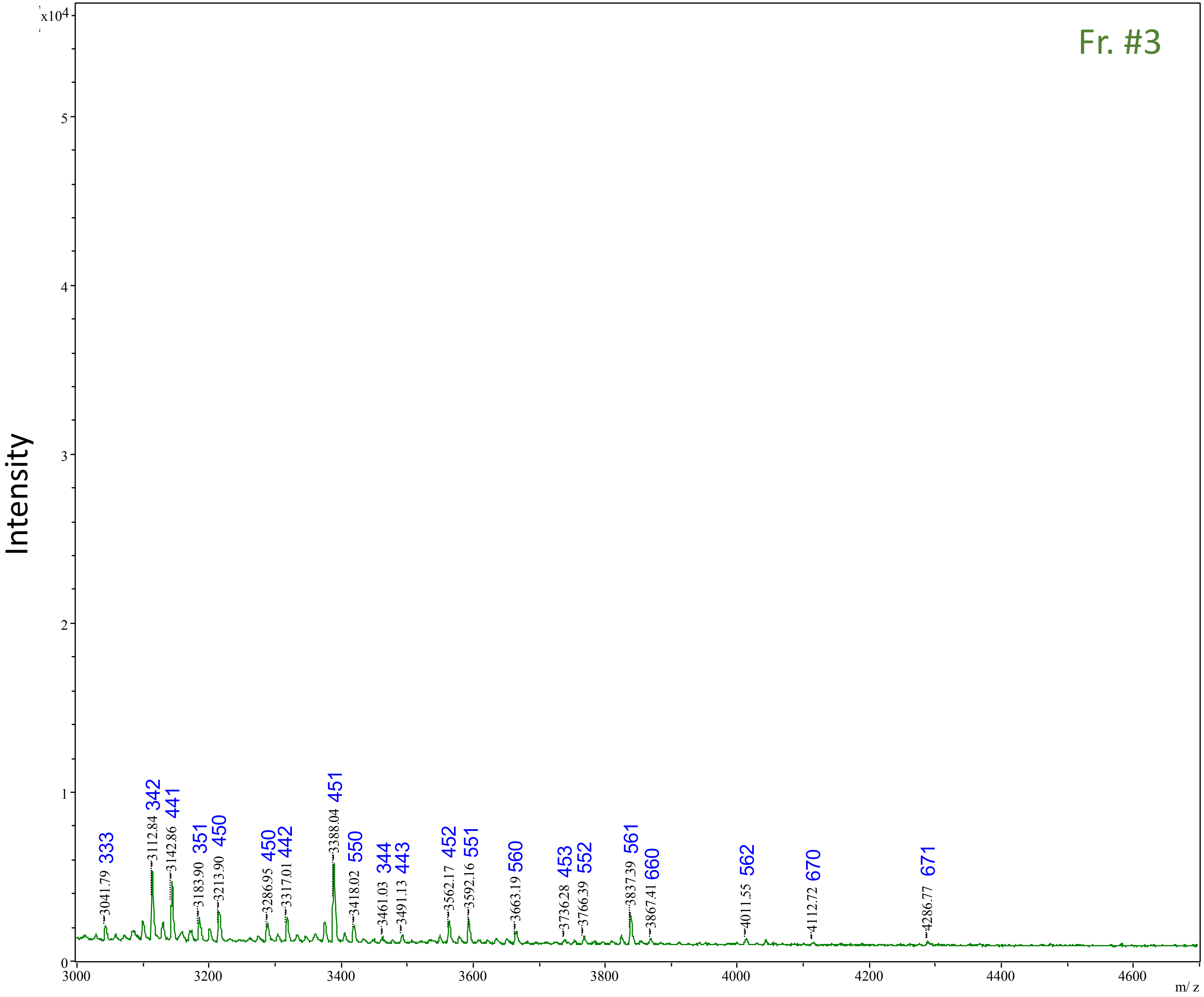

Fig. S1-7. Togayachi et al.

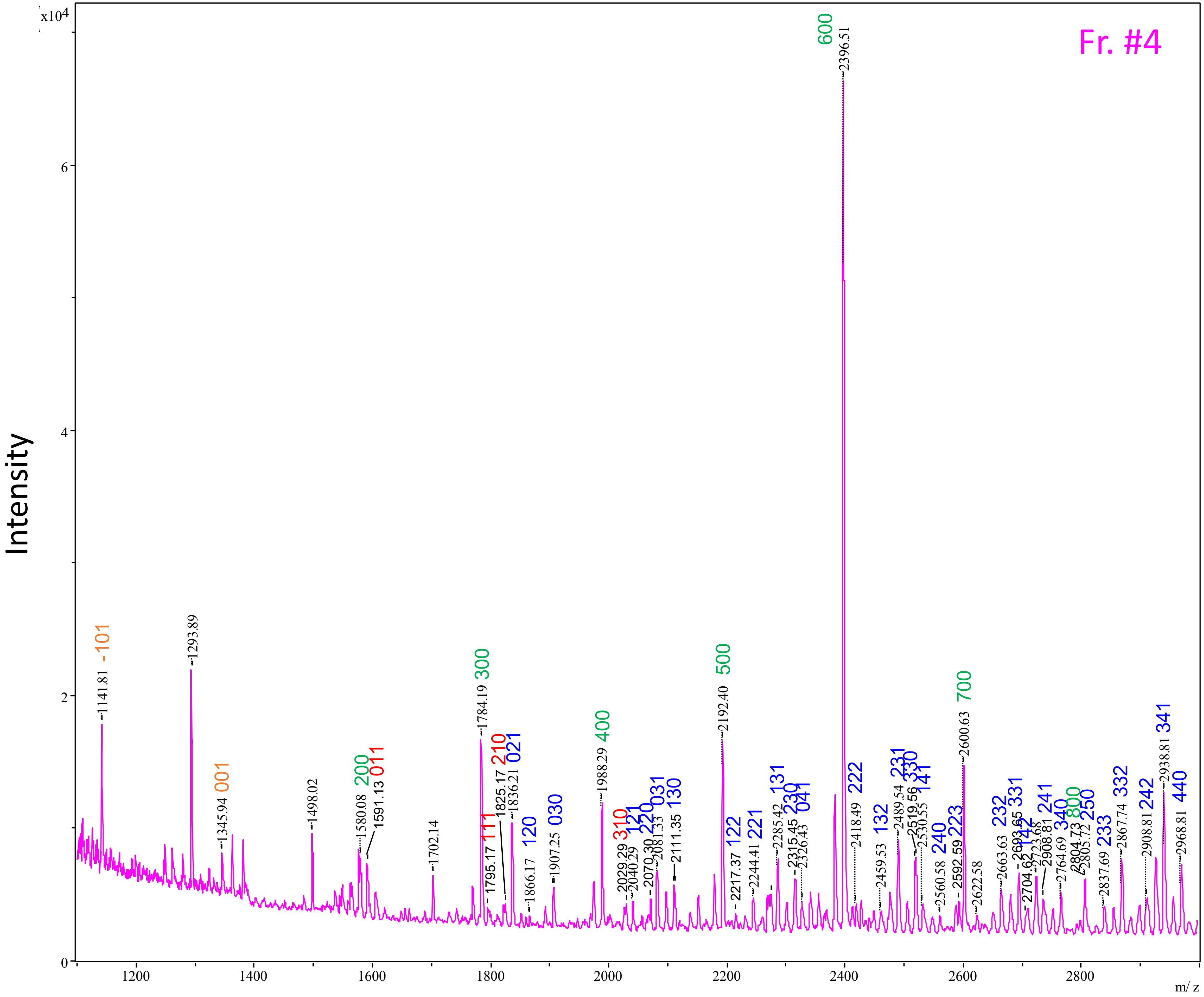

Fig. S1-8. Togayachi et al.

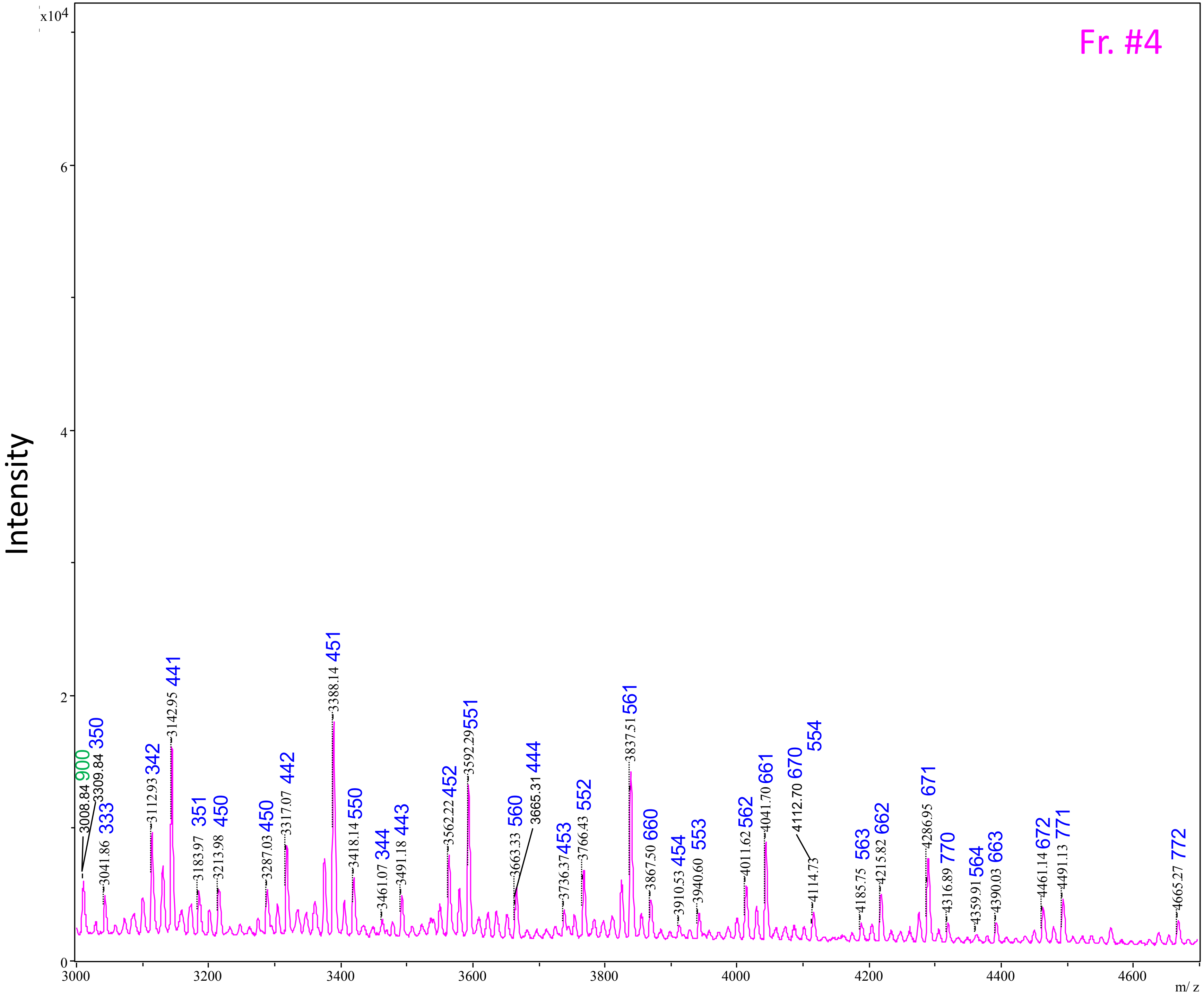

Fig. S1-9. Togayachi et al.

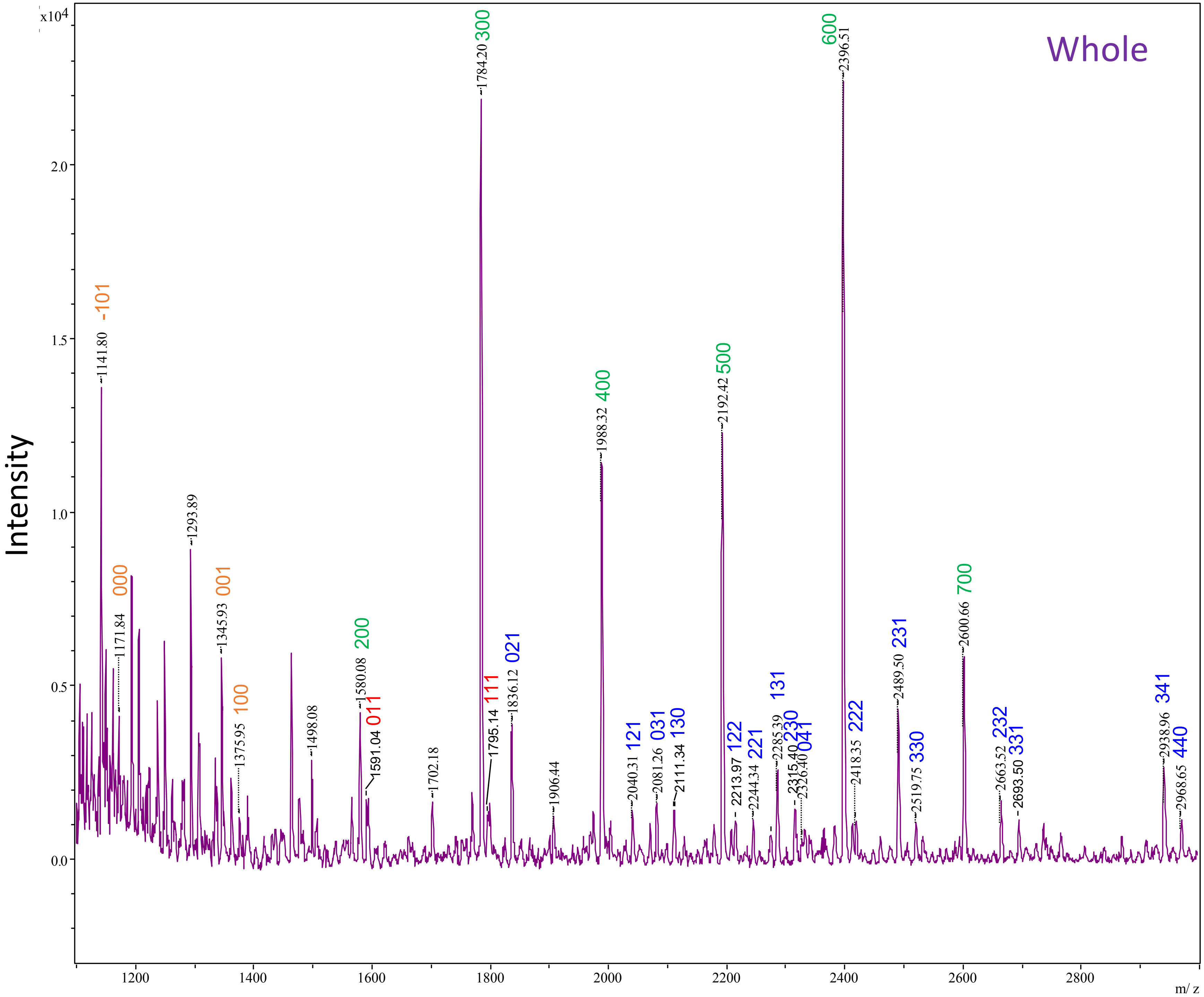

Fig. S1-10. Togayachi et al.

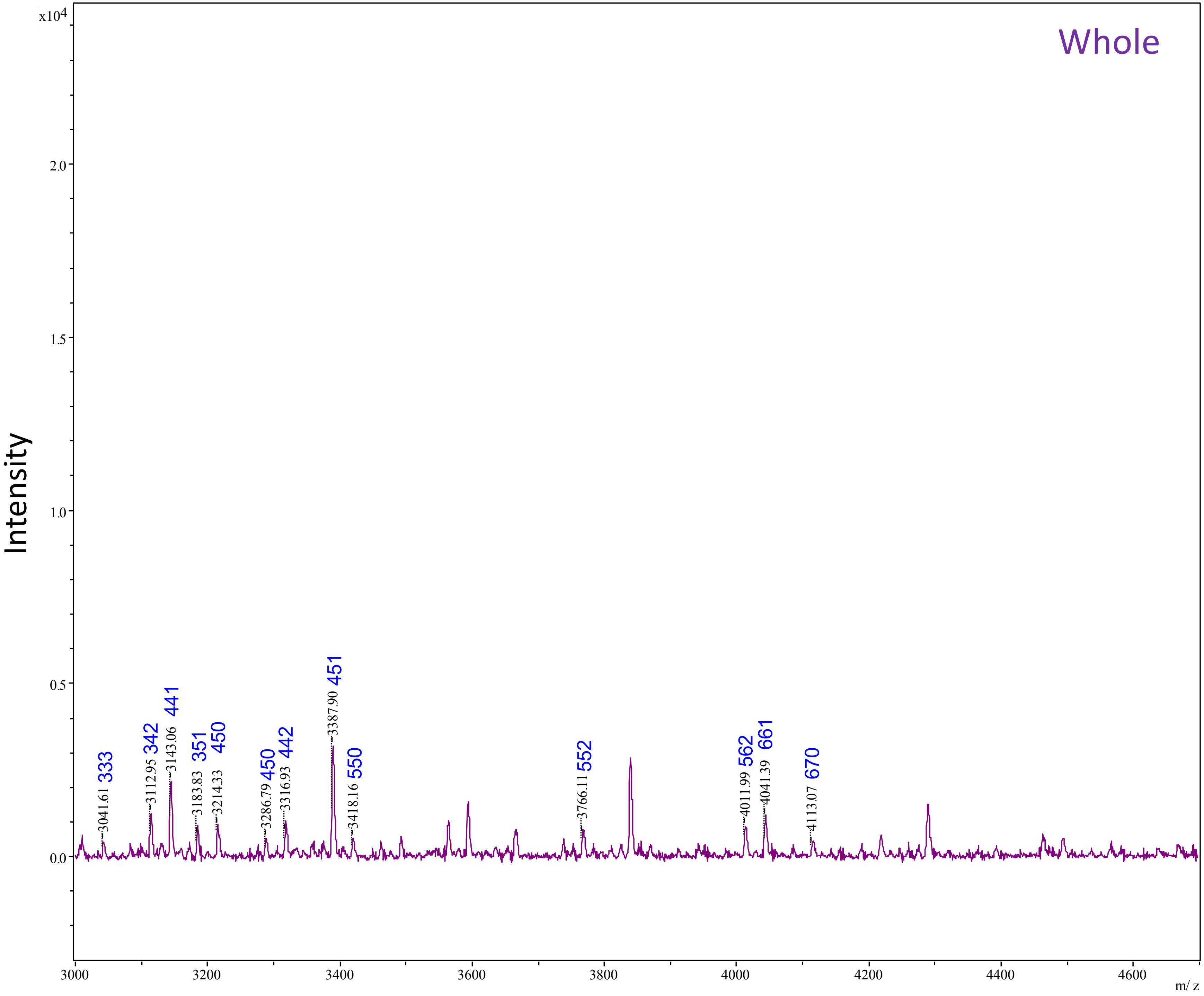

Fig. S1-11. Togayachi et al.

Fr. #1-5773

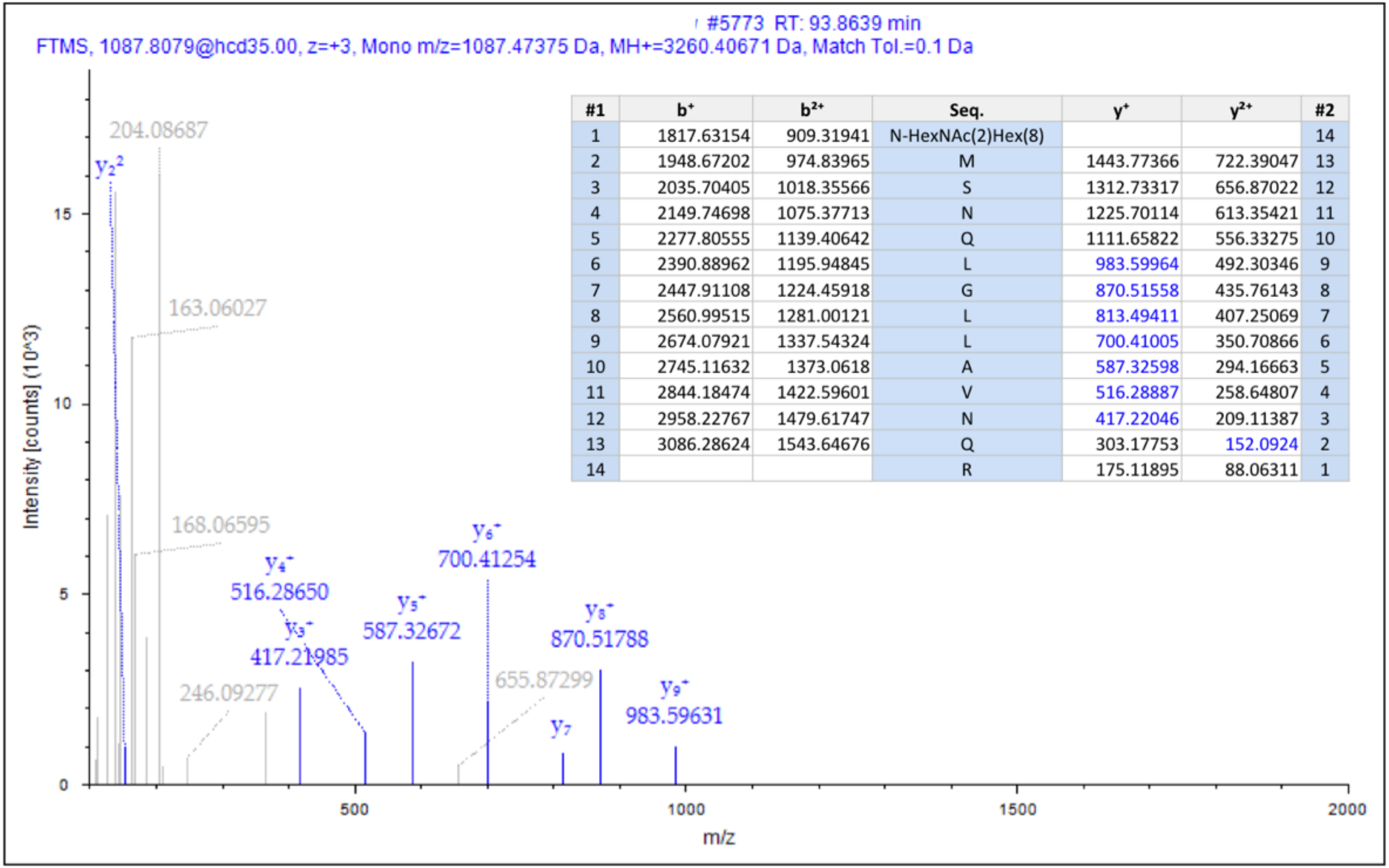

#1	b^+	b^{2+}	Seq.	y^+	y^{2+}	#2
1	1817.63154	909.31941	N-HexNAc(2)Hex(8)			14
2	1948.67202	974.83965	M	1443.77366	722.39047	13
3	2035.70405	1018.35566	S	1312.73317	656.87022	12
4	2149.74698	1075.37713	N	1225.70114	613.35421	11
5	2277.80555	1139.40642	Q	1111.65822	556.33275	10
6	2390.88962	1195.94845	L	983.59964	492.30346	9
7	2447.91108	1224.45918	G	870.51558	435.76143	8
8	2560.99515	1281.00121	L	813.49411	407.25069	7
9	2674.07921	1337.54324	L	700.41005	350.70866	6
10	2745.11632	1373.0618	A	587.32598	294.16663	5
11	2844.18474	1422.59601	V	516.28887	258.64807	4
12	2958.22767	1479.61747	N	417.22046	209.11387	3
13	3086.28624	1543.64676	Q	303.17753	152.0924	2
14			R	175.11895	88.06311	1

Fig. S2-1. Togayachi et al.

Fr. #2-3807

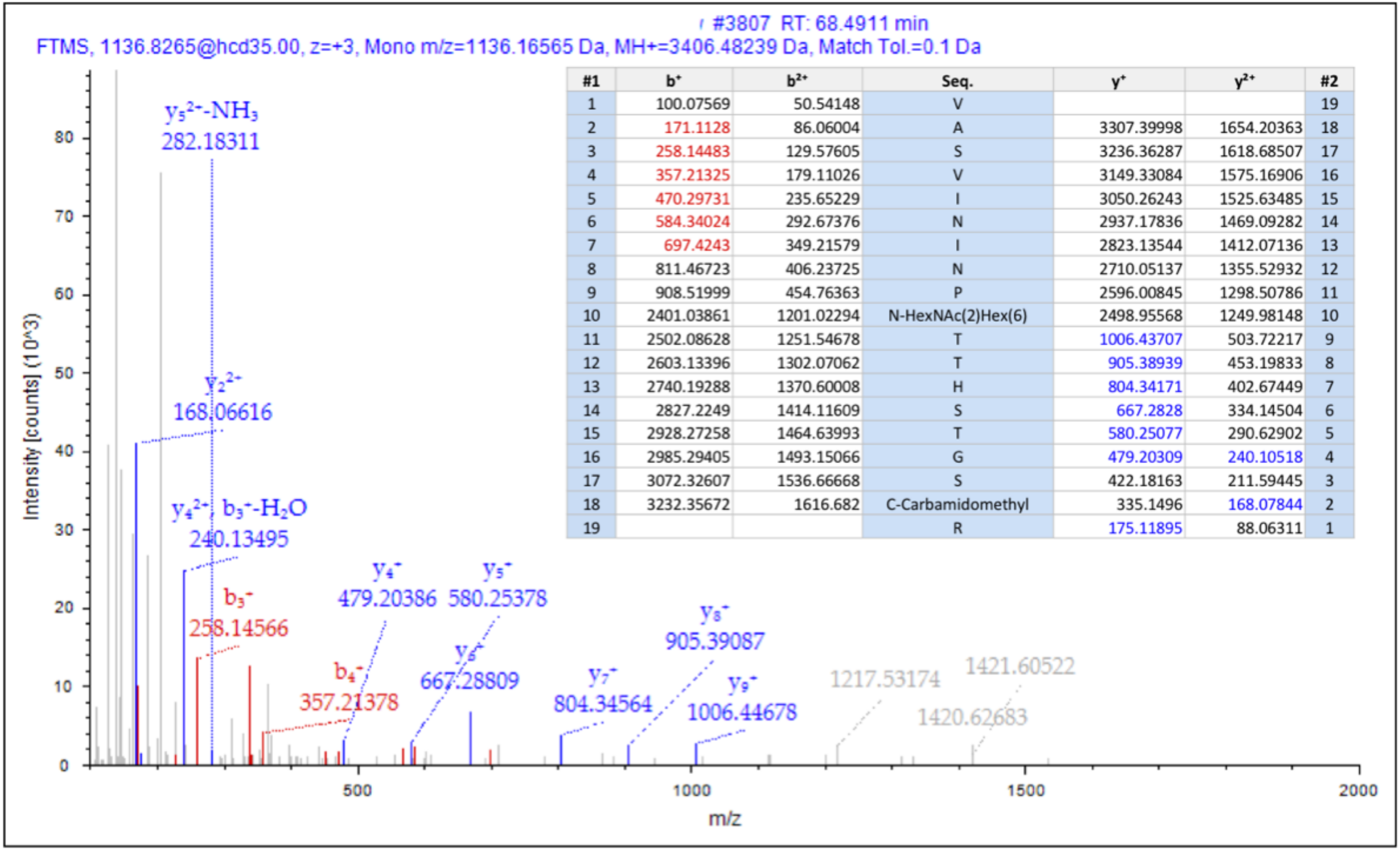

#1	b^+	b^{2+}	Seq.	y^+	y^{2+}	#2
1	100.07569	50.54148	V			19
2	171.1128	86.06004	A	3307.39998	1654.20363	18
3	258.14483	129.57605	S	3236.36287	1618.68507	17
4	357.21325	179.11026	V	3149.33084	1575.16906	16
5	470.29731	235.65229	I	3050.26243	1525.63485	15
6	584.34024	292.67376	N	2937.17836	1469.09282	14
7	697.4243	349.21579	I	2823.13544	1412.07136	13
8	811.46723	406.23725	N	2710.05137	1355.52932	12
9	908.51999	454.76363	P	2596.00845	1298.50786	11
10	2401.03861	1201.02294	N-HexNAc(2)Hex(6)	2498.95568	1249.98148	10
11	2502.08628	1251.54678	T	1006.43707	503.72217	9
12	2603.13396	1302.07062	T	905.38939	453.19833	8
13	2740.19288	1370.60008	H	804.34171	402.67449	7
14	2827.2249	1414.11609	S	667.2828	334.14504	6
15	2928.27258	1464.63993	T	580.25077	290.62902	5
16	2985.29405	1493.15066	G	479.20309	240.10518	4
17	3072.32607	1536.66668	S	422.18163	211.59445	3
18	3232.35672	1616.682	C-Carbamidomethyl	335.1496	168.07844	2
19			R	175.11895	88.06311	1

Fig. S2-2. Togayachi et al.

Fr. #2-4729

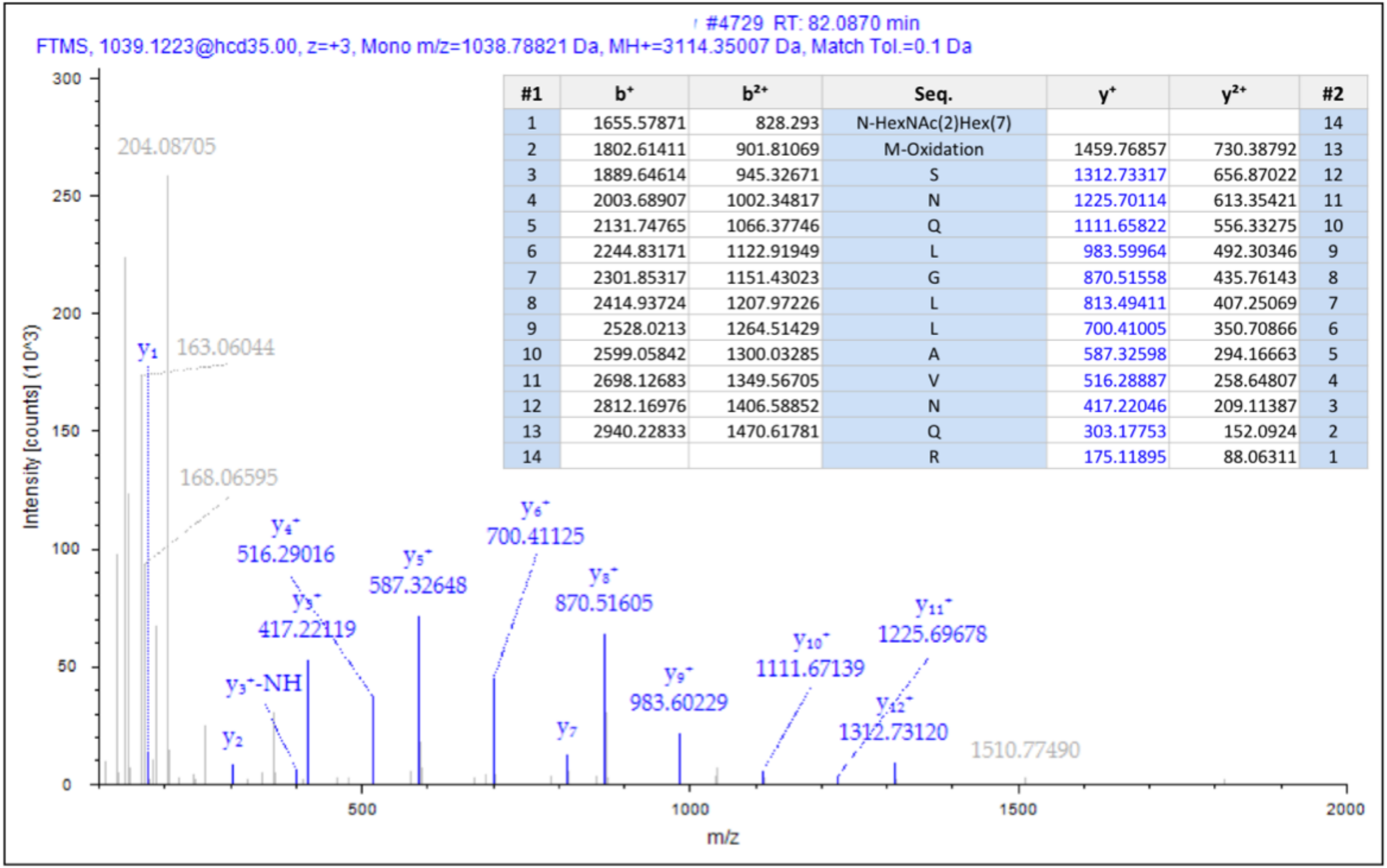

#1	b^+	b^{2+}	Seq.	y^+	y^{2+}	#2
1	1655.57871	828.293	N-HexNAc(2)Hex(7)			14
2	1802.61411	901.81069	M-Oxidation	1459.76857	730.38792	13
3	1889.64614	945.32671	S	1312.73317	656.87022	12
4	2003.68907	1002.34817	N	1225.70114	613.35421	11
5	2131.74765	1066.37746	Q	1111.65822	556.33275	10
6	2244.83171	1122.91949	L	983.59964	492.30346	9
7	2301.85317	1151.43023	G	870.51558	435.76143	8
8	2414.93724	1207.97226	L	813.49411	407.25069	7
9	2528.0213	1264.51429	L	700.41005	350.70866	6
10	2599.05842	1300.03285	A	587.32598	294.16663	5
11	2698.12683	1349.56705	V	516.28887	258.64807	4
12	2812.16976	1406.58852	N	417.22046	209.11387	3
13	2940.22833	1470.61781	Q	303.17753	152.0924	2
14			R	175.11895	88.06311	1

Fig. S2-3. Togayachi et al.

Fr. #3-1949

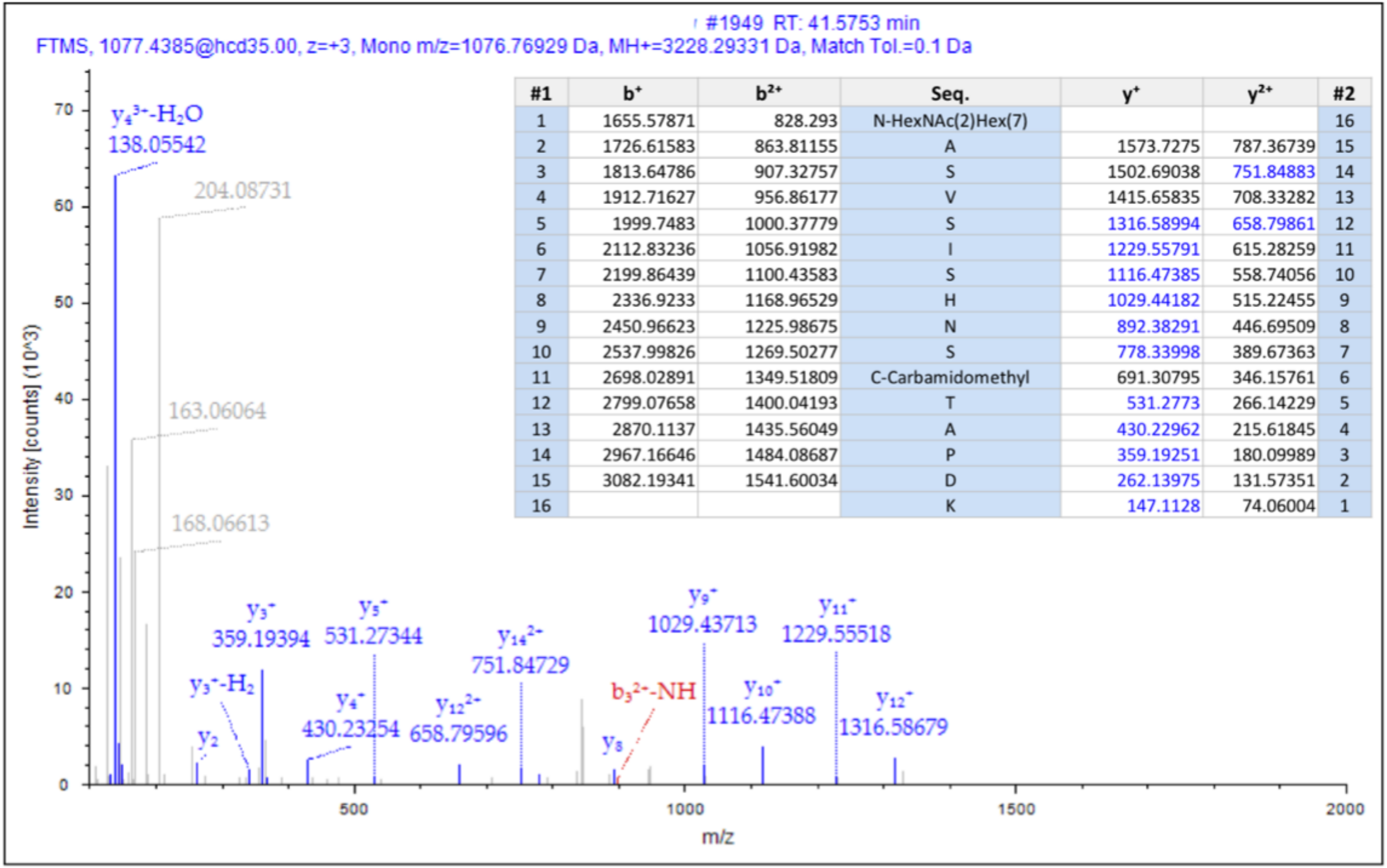

#1	b^+	b^{2+}	Seq.	y^+	y^{2+}	#2
1	1655.57871	828.293	N-HexNAc(2)Hex(7)			16
2	1726.61583	863.81155	A	1573.7275	787.36739	15
3	1813.64786	907.32757	S	1502.69038	751.84883	14
4	1912.71627	956.86177	V	1415.65835	708.33282	13
5	1999.7483	1000.37779	S	1316.58994	658.79861	12
6	2112.83236	1056.91982	I	1229.55791	615.28259	11
7	2199.86439	1100.43583	S	1116.47385	558.74056	10
8	2336.9233	1168.96529	H	1029.44182	515.22455	9
9	2450.96623	1225.98675	N	892.38291	446.69509	8
10	2537.99826	1269.50277	S	778.33998	389.67363	7
11	2698.02891	1349.51809	C-Carbamidomethyl	691.30795	346.15761	6
12	2799.07658	1400.04193	T	531.2773	266.14229	5
13	2870.1137	1435.56049	A	430.22962	215.61845	4
14	2967.16646	1484.08687	P	359.19251	180.09989	3
15	3082.19341	1541.60034	D	262.13975	131.57351	2
16			K	147.1128	74.06004	1

Fig. S2-4. Togayachi et al.

Fr. #4-1978

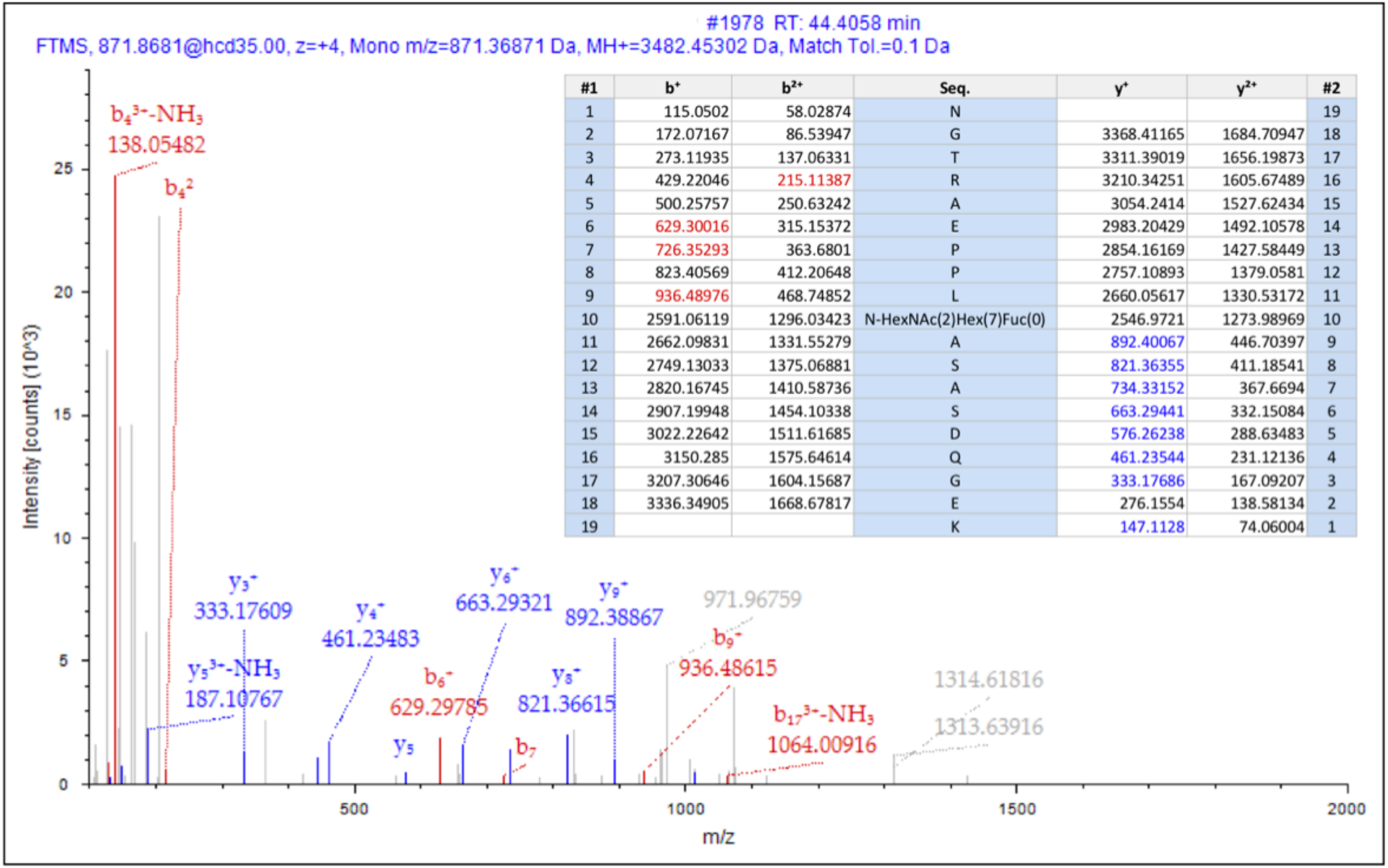

#1	b^+	b^{2+}	Seq.	y^+	y^{2+}	#2
1	115.0502	58.02874	N			19
2	172.07167	86.53947	G	3368.41165	1684.70947	18
3	273.11935	137.06331	T	3311.39019	1656.19873	17
4	429.22046	215.11387	R	3210.34251	1605.67489	16
5	500.25757	250.63242	A	3054.2414	1527.62434	15
6	629.30016	315.15372	E	2983.20429	1492.10578	14
7	726.35293	363.6801	P	2854.16169	1427.58449	13
8	823.40569	412.20648	P	2757.10893	1379.0581	12
9	936.48976	468.74852	L	2660.05617	1330.53172	11
10	2591.06119	1296.03423	N-HexNAc(2)Hex(7)Fuc(0)	2546.9721	1273.98969	10
11	2662.09831	1331.55279	A	892.40067	446.70397	9
12	2749.13033	1375.06881	S	821.36355	411.18541	8
13	2820.16745	1410.58736	A	734.33152	367.6694	7
14	2907.19948	1454.10338	S	663.29441	332.15084	6
15	3022.22642	1511.61685	D	576.26238	288.63483	5
16	3150.285	1575.64614	Q	461.23544	231.12136	4
17	3207.30646	1604.15687	G	333.17686	167.09207	3
18	3336.34905	1668.67817	E	276.1554	138.58134	2
19			K	147.1128	74.06004	1

Fig. S2-5. Togayachi et al.

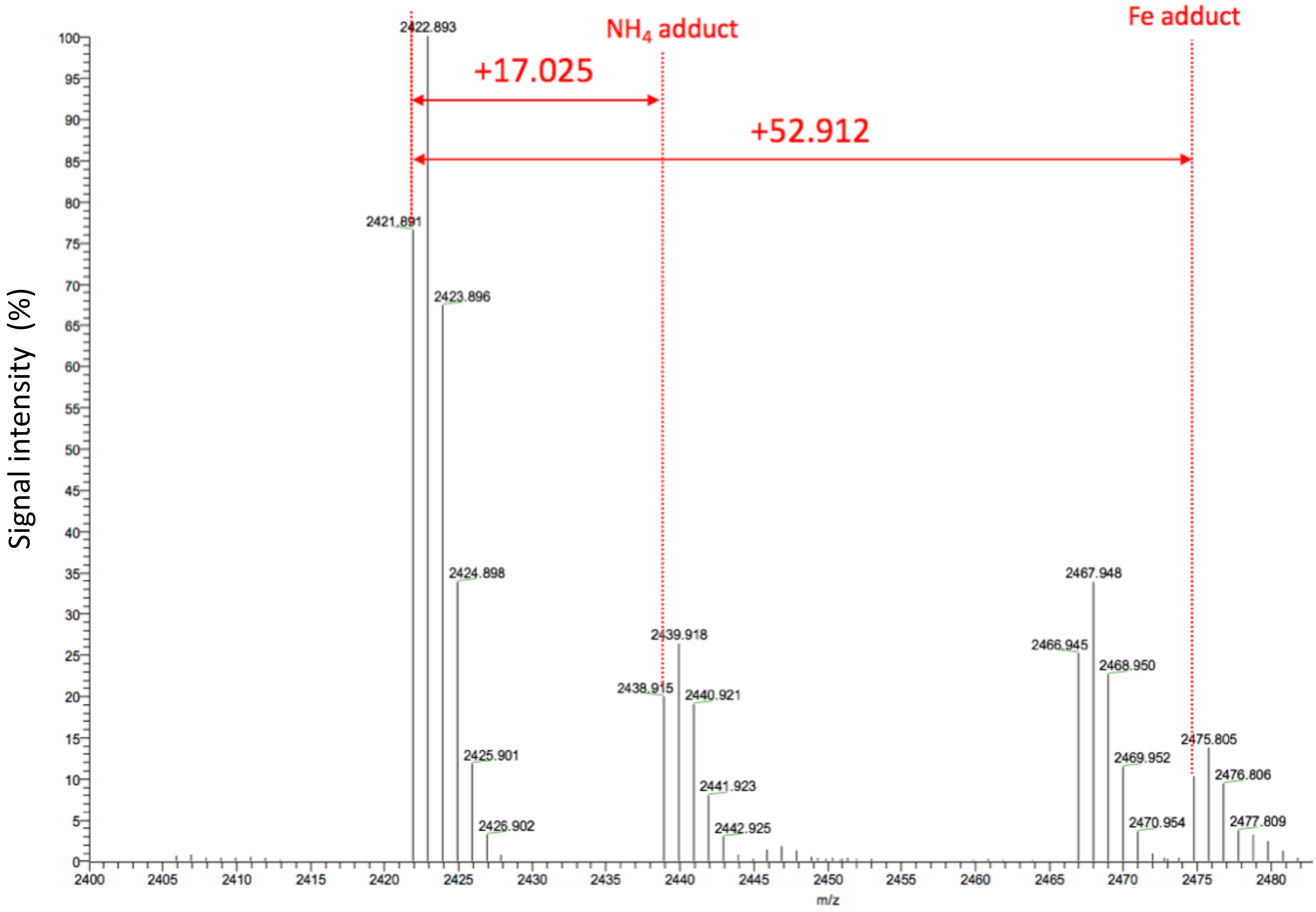

Fig. S3. Togayachi et al.

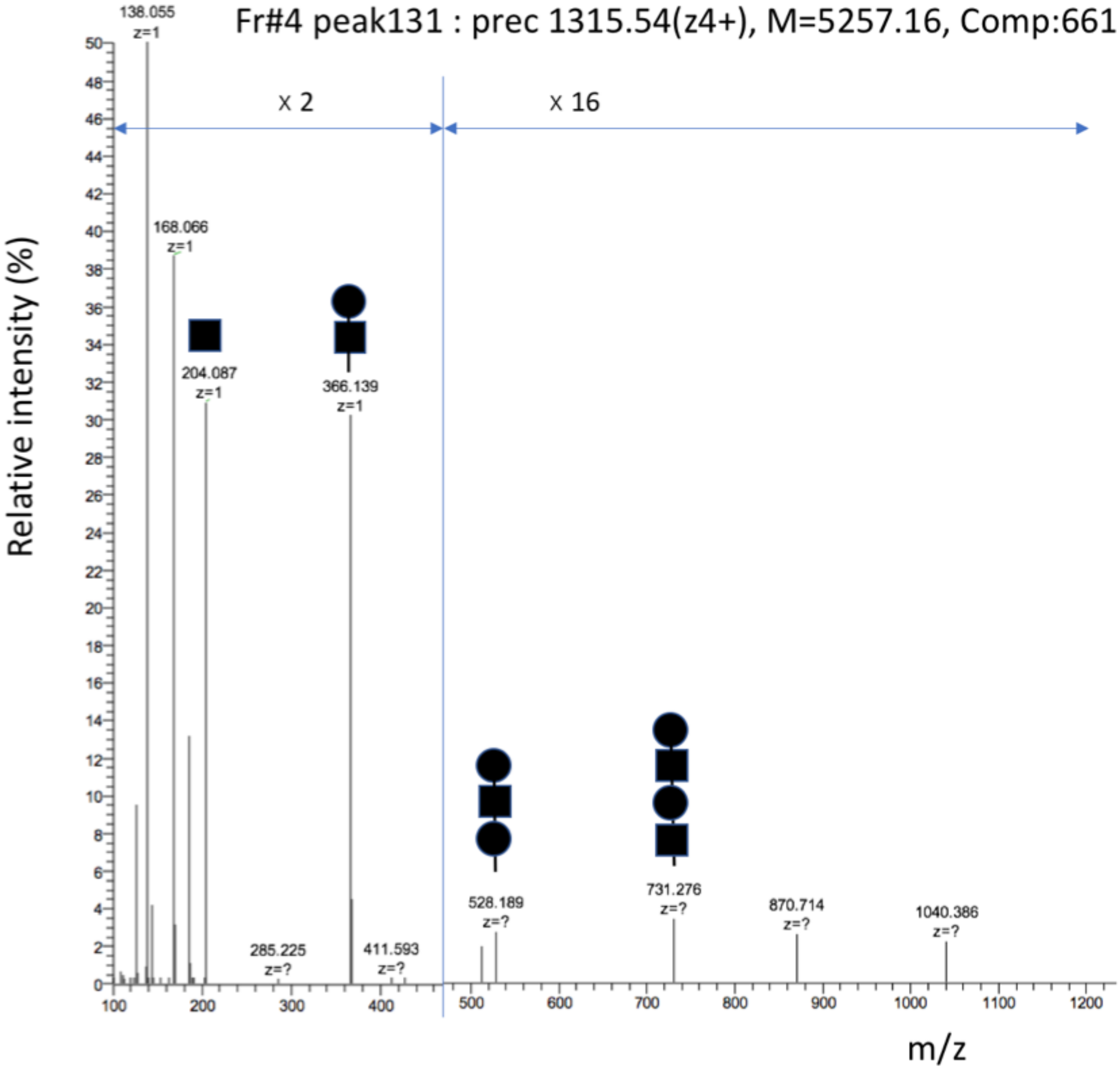

Fig. S4. Togayachi et al.
